# A re-annotation of the *Anopheles darlingi* mobilome

**DOI:** 10.1590/1678-4685-GMB-2017-0300

**Published:** 2019-01-21

**Authors:** Jose Francisco Diesel, Mauro F. Ortiz, Osvaldo Marinotti, Ana Tereza R. Vasconcelos, Elgion L. S. Loreto

**Affiliations:** 1 Universidade Federal de Santa Maria Universidade Federal de Santa Maria Departamento de Bioquímica e Biologia Molecular Santa MariaRS Brazil Departamento de Bioquímica e Biologia Molecular, Universidade Federal de Santa Maria, Santa Maria, RS, Brazil; 2 Universidade Federal do Rio Grande do Sul Universidade Federal do Rio Grande do Sul Programa de Pós-Graduação de Genética e Biologia Molecular Porto AlegreRS Brazil Programa de Pós-Graduação de Genética e Biologia Molecular, Universidade Federal do Rio Grande do Sul, Porto Alegre, RS, Brazil; 3 University of California University of California Department of Molecular Biology and Biochemistry IrvineCA USA Department of Molecular Biology and Biochemistry, University of California at Irvine, Irvine, CA, USA; 4 Laboratório de Bioinformática do Laboratório Nacional de Computação Científica Laboratório de Bioinformática do Laboratório Nacional de Computação Científica PetrópolisRJ Brazil Laboratório de Bioinformática do Laboratório Nacional de Computação Científica, Petrópolis, RJ, Brazil

**Keywords:** Transposable elements, transposons, mosquitoes, evolvability.

## Abstract

The mobilome, portion of the genome composed of transposable elements (TEs), of *Anopheles darlingi* was described together with the genome of this species. Here, this mobilome was revised using similarity and *de novo* search approaches. A total of 5.6% of the *A. darlingi* genome is derived of TEs. Class I *gypsy* and c*opia* were the most abundant superfamilies, corresponding to 22.36% of the mobilome. Non-LTR elements of the *R1* and *Jockey* superfamilies account for 11% of the TEs. Among Class II TEs, the *mariner* superfamily is the most abundant (16.01%). Approximately 87% of the *A. darlingi* mobilome consist of short, truncated and/or degenerated copies of TEs. Only three retrotransposons, two belonging to *gypsy* and one to *copia* superfamilies, are putatively active elements. Only one Class II element, belonging to the *mariner* superfamily, is putatively active, having 12 copies in the genome. The TE landscape of *A. darlingi* is formed primarily by degenerated elements and, therefore, somewhat stable. Future applications of TE-based vectors for genetic transformation of *A. darlingi* should take into consideration *mariner* and *piggyBac* transposons, because full length and putatively active copies of these elements are present in its genome.

## Introduction

The mobilome is the complete set of mobile genetic elements in a genome. In eukaryotes, it is constituted mainly by transposable elements (TEs) (Siefert, 2009), comprising about 45% of the human genome, 20% of *D. melanogaster*, and more than 50% of the maize genome (SanMiguel *et al.*, 1996; Lander *et al.*, 2001; Kaminker *et al.*, 2002). Transposable elements are drivers of evolution, as a source of genetic variability, generally by promoting chromosome rearrangements, mutations in the coding or regulatory regions of genes, domestication and epigenetic alterations (reviewed in Hua-Van *et al.*, 2011).

TEs are classified as autonomous elements when they are able to produce the enzymes necessary for their own mobilization, or as non-autonomous when they require enzymes produced by related autonomous elements for that activity. TEs have also been classified into two classes, namely RNA-mediated (Class-I) and DNA-mediated (Class-II) elements, according to their transposition mode. TEs often occur as remains or relics of old elements, which are not mobilizable any more. The combination of active, mobilizable, and remnants of TEs constitutes the TE landscape of a genome, which is characteristic of a species. For example, in humans, the LINE-1/L1-element is the only element that is presently active, while in *Drosophila melanogaster* 30% of the TEs are full length and potentially active (Kaminker *et al.*, 2002). Closely related species can display distinct TE contents. For instance, TE contents vary from 2.7 to 23% among the genomes of 12 *Drosophila* species (Clark *et al.*, 2007), and among *Anopheles* species it varies from 1.98 to 17.78% (Neafsey *et al.*, 2015). Yet, a full, integral insight into the mobilome is not provided just by the proportion of a genome occupied by TEs and their classification. Also important is the identification of full length, putatively active elements. This aspect is particularly important for organisms that are potential candidates for genetic manipulation using transposon-based transgenic technologies. In these cases, the characterization of full length and active transposable elements is fundamental to estimate genomic stability and biosafety of the proposed products (Terenius *et al.*, 2008). In genetically transformed organisms, the presence of active endogenous TEs similar to the one(s) used in the transformation vector(s) might interfere with the efficiency of transgene integration and transgene stability due to cross mobilization (Arensburger *et al.*, 2011).

The classification and annotation of TEs is always a challenging task due to their remarkable diversity within and among genomes. TE copies recently inserted into a genome show low sequence variability, though with time passing, copies accumulate mutations, deletions, and/or insertions, becoming decayed TE remnants (Hua-Van *et al.*, 2011; Hoen *et al.*, 2015). Two main approaches are currently used for TEs identification and annotation. Homology-based methods search for sequences similar to known TEs compiled in databases. The *de novo* approach is based on the search for repetitiveness and structural signatures normally found in TEs (Hoen *et al.*, 2015). New tools for mobilome scrutiny, exploration, and annotation warrant the re-analysis of previously described genomes (Kaminker *et al.*, 2002). Fernández-Medina *et al.* (2011) who re-analyzed the mobilome of *A. gambiae*, found new TEs, described complete and potentially active elements, and characterized additional deleted, mutated, and probably inactive copies.

*Anopheles darlingi* is the principal Neotropical malaria vector, responsible for more than a million malaria cases per year (Oliveira-Ferreira *et al.*, 2010). The genome of this mosquito was sequenced, annotated, and its mobilome described (Marinotti *et al.*, 2013). In that study, TEs were annotated applying a homology-based method, using a “home-made” TE database. *De novo* search was used only to find MITEs and SINEs. In the present study, the Repbase database was used for a homology-based search, and the Repeatscout program was used for *de novo* searches. These improved approaches allowed us to advance our knowledge of the *A. darlingi* mobilome, and to revise the number and annotation of the identified TEs in its genome*.*

## Material and Methods

**Figure 1:**
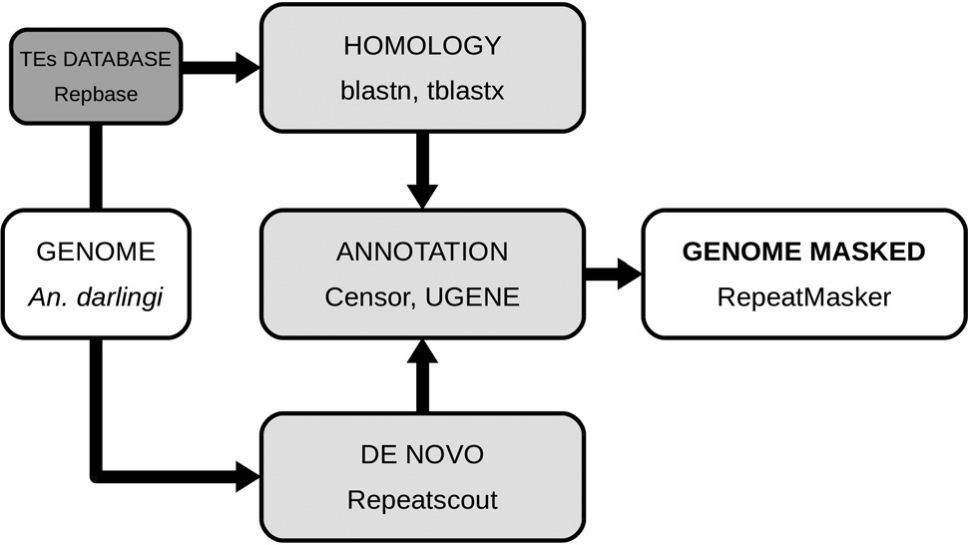
Flowchart depicting the pipeline implemented in this study for analysis and annotation of the *An. darlingi* mobilome. The assembled genome (ADMH02000000) was screened for TEs by similarity-based approach, using the Repbase database by Blastn and tblastx. In parallel, the genome was screened *de novo* by using Repeatscout. Redundancies were removed and TEs were classified using Censor with the Repbase database. Also, a manual annotation was performed, using UGENE, with emphasis on TIRs, LTRs, TSDs and ORFs. The content of TEs in the genome was calculated using RepeatMasker.

*A. darlingi* transposable elements were identified following the pipeline shown in Figure 1. Blastn and tblastx (Altschul *et al.*, 1997) were used to find similarities of *A. darlingi* genome sequences (GenBank accession number ADMH02000000) (Marinotti *et al.*, 2013) with the TEs references of the Repbase database (version 18.01) (Jurka *et al.*, 2005), considering e-values < 10e-10 as a cutoff to define a Blast “hit”. Redundancies representing hits at overlapping genomic positions, for different TEs, were considered as one hit for further analyses. Each target was expanded 5 kb on each side and searched for TIRs, LTR, TSD and conserved ORFs with the UGENE platform (Okonechnikov *et al.*, 2012). The Censor software (Kohany *et al.*, 2006) implemented with the Repbase database was used for the classification and annotation of TEs. For *de novo* searches, Repeatscout version 1.0.0 software (Price *et al.*, 2005) was used. The obtained sequences were analyzed using UGENE to look for characteristics described previously (TIRs, LTRs, etc.) and to classify these transposable elements. After obtaining a full library by similarities and *de novo* searches, the genome was masked to determine the number of transposable elements using RepeatMasker (with *-no_is -nolow* options) (Smit *et al.*, 2016). TEs were classified using Repbase (DNA, ERV, LTR, Non-LTR), and their number of copies and % of genome were calculated.

Many short retrieved sequences show similarities with known TEs sequences. These short sequences are mentioned in the text as “hits” and classified as belonging to the superfamily corresponding to the best blast hit (lowest e-value). Sequences longer than 200 bp were manually curated with the UGENE platform for the annotation of ORFs, TIR, LTRs, and TSDs. All ORFs were analyzed by Blastp, and those showing similarities with TEs-encoded proteins were manually annotated. The sequences with typical TE characteristics were designated as identifiable TEs (ITEs) and classified as: (1) complete elements - containing TIR (or LTRs) and complete ORFs; (2) degenerated – most often contain TIRs (or LTRs), however, the ORFs present mutations and deletions; (3) truncated - these elements show large deletions; (4) MITEs – short elements, having TIRs. When possible, sequences were assigned into families using Repbase; otherwise they were described as Unknown.

The repeat landscape of TEs found in the *A. darlingi* genome was constructed with the full TE dataset obtained using the RepeatMasker tool.

## Results

The TEs identified in this study correspond to 5.6% of the *A. darlingi* genome (Table 1). Class I elements correspond to 2.64% and Class II to 2.44% of the genome. For Class I, the superfamilies *gypsy* and c*opia* were the most abundant. Together, these superfamilies account for 22.36% of all TEs. Non-LTR elements of *R1* and *Jockey* superfamilies compound, together, 11% of all TEs. The *mariner* superfamily is the most abundant among the Class II elements (16.01%). Endogenous retroviruses (ERVs) correspond to 8.23% of the TEs.

**Table 1:** Sequences showing significant hits with TEs identified in *Anopheles darlingi* genome.

Class	Order	Superfamily	Hit number	Occupied size (bp)	TE %	Genome %
I (2.64%)	LTR (1.40%)	Gypsy	18007	1304425	11.27	0.75
		Copia	17890	1283868	11.09	0.74
		BEL	1262	114186	0.98	0.06
		DIRS	740	47004	0.41	0.03
		Others	2589	159289	1.37	0.09
	Non-LTR (1.25%)	R1	7744	654295	5.65	0.37
		Jockey	7685	614572	5.31	0.35
		Tx1	4074	302983	2.62	0.17
		L1	3944	256751	2.22	0.15
		SINE2/tRNA	2645	199096	1.72	0.11
		Others	7100	589270	5.09	0.34
II (2.44%)		Mariner/Tc1	11871	1852562	16.01	1.06
		hAT	11167	723088	6.23	0.41
		EnSpm/CACTA	6961	474890	4.10	0.27
		Polinton	4207	268339	2.32	0.15
		Helitron	2552	186142	1.61	0.11
		Others	21620	1580047	13.65	0.91
ERVs (0.53%)		ERV1	5835	441984	3.82	0.25
		ERV2	5562	383990	3.32	0.22
		ERV3	670	48140	0.41	0.03
		ERV4	68	4117	0.03	0.01
		Others	1279	83874	0.72	0.04
**TOTAL**			**145472**	**11572912**	**100**	**6.65**
TOTAL*						**5.67**

* without redundancy

A remarkable aspect of the *A. darlingi* mobilome is that it is composed mainly of very short sequences displaying significant similarities with TEs present in the used database. These hits are likely derived from extensively mutated and/or deleted TEs, lacking clearly identifiable TE structural features such as TIRs, LTRs, or TSD, etc.. The sequences containing identifiable TE structural features, ITEs, correspond to only 0.87% of the *A. darlingi* genome (Table 2). This value was obtained by multiplying the size of each element by copy number, which corresponds to 1.5 Mb. The entire *A. darlingi* genome was estimated to be 173.9 Mb (Marinotti *et al.*, 2013). Thirty-six ITE elements were found, 26 belonging to the *mariner* family, one to the *piggyBac* family, one to *kolobok* family, two elements are from *the gypsy* family, one from the *copia* family, and four DNA/Unknown elements (Table 2). Short descriptions and sequences of each element are presented in List S1 of the Supplementary Material.

**Table 2:** Identifiable TEs found in the *An. darlingi* genome.

Seq	TE Name	Superfamily	Copies	Size	Censor Hit	ID(%)	Score	Status	TIRs (bp)	TSD
1	Mariner1-Andl	DNA/Mariner	190	907	Mariner-N2_SIn	71	1769	Degen.	-	TA
2	Mariner2-Andl	DNA/Mariner	32	941	ITmD37D_Ele1	68	1581	Degen.	-	TA
3	Mariner3-Andl	DNA/Mariner	28	1265	Mariner-6_PBa	75	3657	Degen.	-	TA
4	Mariner4-Andl	DNA/Mariner	44	1194	Mariner-2_ACe	79	5637	Truncated	-	TA
5	Mariner5-Andl	DNA/Mariner	91	890	Mariner-2_AEc	71	1376	Degen.	-	TA
6	Mariner6-Andl	DNA/Mariner	30	905	Mariner-30_SIn	77	2180	Degen.	-	TA
7	Mariner7-Andl	DNA/Mariner	99	912	Mariner-1_DF	65	274	Degen.	-	TA
8	Mariner8-Andl	DNA/Mariner	20	1673	AeTango2	65	2023	Truncated	-	TA
9	Mariner9-Andl	DNA/Mariner	10	738	CRMAR	71	1749	MITE	223	TA
10	Mariner10-Andl	DNA/Mariner	2	489	MARINER_CA	72	1371	MITE	22	TA
11	Mariner11-Andl	DNA/Mariner	12	1664	Mariner-8-Dan	68	1317	Put. Active	230	TA
12	Mariner12-Andl	DNA/Mariner	46	1285	ITmD37D_Ele3	65	894	Degen.	30	TA
13	Mariner13-Andl	DNA/Mariner	64	906	Mariner_3_DF	69	1521	Degen.	64	TA
14	Mariner14-Andl	DNA/Mariner	3	1321	Mariner_16_Dan	70	2061	Degen.	33	TA
15	Mariner15-Andl	DNA/Mariner	31	1220	Mariner-16_DAn	70	2390	Degen.	23	TA
16	Mariner16-Andl	DNA/Mariner	3	1471	Mariner-3_DF	70	376	Degen.	25	TA
17	Mariner17-Andl	DNA/Mariner	3	886	Mariner-6_BM	66	597	Degen.	29	TA
18	Mariner18-Andl	DNA/Mariner	42	1268	Tc1-1_TCa	68	331	Degen.	215/225	TA
19	Mariner19-Andl	DNA/Mariner	9	792	Mariner-6_BM	65	539	MITE	17	TA
20	Mariner20-Andl	DNA/Mariner	49	759	Tx_mos	65	577	MITE	25	TA
21	Mariner21-Andl	DNA/Mariner	1	796	MARINER_CA	71	894	MITE	30	TA
22	Mariner22-Andl	DNA/Mariner	2	1755	MARINER_CA	68	1673	Degen.	25	TA
23	Mariner23-Andl	DNA/Mariner	200	1212	Tx_mos	63	532	Degen.	-	TA
24	Mariner24-Andl	DNA/Mariner	100	394	-	-	-	Degen.	-	TA
25	Mariner25-Andl	DNA/Mariner	100	328	-	-	-	Degen.	-	TA
26	Mariner26-Andl	DNA/Mariner	21	702	Mariner-58_Ccri	-	-	MITE	28	TA
27	DNAUnknown-Andl1	DNA/Unknown	100	555				Degen.		
28	DNAUnknown-Andl2	DNA/Unknown	100	234	-	-	-	Degen.	-	-
29	DNAUnknown-Andl3	DNA/Unknown	60	514	-	-	-	Degen.	-	-
30	DNAUnknown-Andl4	DNA/Unknown	40	478	-	-	-	Degen.	-	TTAA
31	PiggyBac1_Andl	DNA/piggyBac	27	2954	piggyBac-1_DBi	67	2143	Truncated	19	TTAA
32	Kolobok1-Andl	DNA/Kolobok	47	751	Kolobok-N1_Dan	76	349	Degen.	14	TTAA
33	Helitron1_Andl	DNA/Helitron	85	1327	Helitron-2_DBp	76	275	Degen.	-	A/T
34	Gypsy1_Andl	LTR/Gypsy	1	5366	Gypsy-625_AA-I	67	7288	Put. Active	210/208	-
35	Gypsy2_Andl	LTR/Gypsy	1	4325	GYPSY36-I_AG	68	6928	Put. Active	167/166	-
36	Copia1_Andl	LTR/Copia	1	4294	Copia-70_AA-I	63	2624	Put. Active	184/202	-

*mariner* elements are predominantly degenerated, and 17 elements were classified with this status. The copy number of these degenerated *mariner* elements range from 2 to 200 copies, and their sizes vary from 1755 to 889 bp. Six *mariner* elements were classified as MITEs, ranging from 489 to 796 bp, and are represented by 1 to 49 copies. Two *mariner* elements were classified as truncated. One putatively active *mariner* element was identified. It is represented by 12 copies in the genome, has a length of 1664 bp, and long TIRs with 230 bp.

Four degenerated elements were classified as DNA/Unknown because they have characteristics of class II elements, but their similarities with known TEs are not high enough for their classification into known families. The copy number of these elements is generally high, ranging from 40 to more than 100 copies in the genome. Their sizes range from 234 to 555 bp, suggesting they are truncated elements.

A truncated *piggyBac* element of 2954 bp with a 19 bp TIR was found, with a total of 27 copies. Degenerated *kolobok* and *helitron* elements were also found, with 47 and 85 copies, respectively.

Only three Class I putatively active ITEs were found. Two elements are from the *gypsy* family, having lengths of 5366 and 4325 bp, with LTRs of 210 and 167 bp, respectively. The third element, belonging to the *copia* family, is 4294 bp in length and has LTRs with 184/202 bp. Only one copy of each of these retrotransposons was found in the *A. darlingi* genome*.*

**Figure 2:**
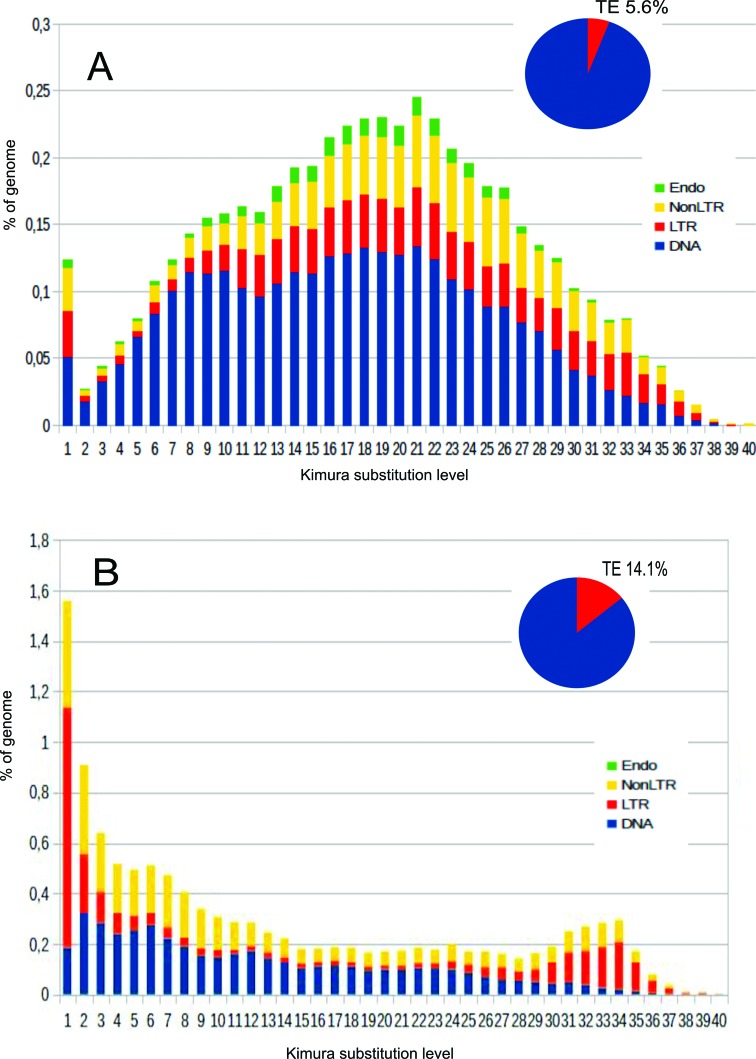
Comparison of TE landscapes of *A. darlingi* (A) and *A. gambiae* (B). The pie charts show the proportion of the genome that is occupied by TEs (The value represented by the blue slice corresponds to portion that is not TEs). In the bar charts, the ordinate illustrates the percentage of the genome occupied by each TE, and the abscissa illustrates the genetic divergence from the consensus (Kimura substitution level- K2P x 100) observed among copies of each TE. Each TE superfamily is color coded. The landscapes were generated using www.repeatmasker.org/genomicDatasets/RMGenomicDatasets.html..

Analysis of nucleotide divergence among the different copies of the found elements allowed to depict a general landscape of the mobilome. The level of Kimura substitution observed among the analyzed sequences is generally high (Figure 2). Few copies of the elements are well conserved, indicating that only few elements are active or have been recently mobilized or duplicated. So the mobilome of *A. darlingi* is constituted mainly of remains of degraded and ancient elements. In comparison, the proportion of copies showing high similarities is greater in *A. gambiae* than in *A. darlingi* (Figure 2), suggesting the presence of active or more recently mobilized elements in *A. gambiae*.

## Discussion

The results of this study show that a higher proportion of the *A. darlingi* genome (5.61%) is occupied by TEs than the previously reported 2.29% (Marinotti *et al.*, 2013). For other organisms, re-analyses of mobilomes using updated tools and databases also resulted in distinct results. For example, the genomes of *Drosophila* species and *A. gambiae* have been re-analyzed resulting in improved descriptions of their mobilomes (Kaminker *et al.*, 2002; Fernández-Medina *et al.*, 2011, Neafsey *et al.*, 2015). Neafsey *et al.* (2015) compared the genomes of 16 *Anopheles* species and found that *A. albimanus* was the mosquito with the genome occupied by the lowest proportion of TEs (1.98%), followed by *A. cristyi* (2.81%). The Anopheline species with the highest content of TEs in its genome is *A. gambiae* (17.78%). The authors also reported a direct correlation between genome size and TE content, with species showing smaller genomes tending to have lower TE contents. Similar correlations between genome size and TE content have also been found for other taxa (Sessegolo *et al.*, 2016). The genome size of *A. darlingi* is among the smallest among the sequenced *Anopheles* species, and similar to those found in *A. albimanus* and *A. cristyi* [≈ 180 Mb] (Table S1). While the TE content found in those species is around 2%, our re-analyses increased the *A. darlingi* TE content from 2.29% to 5.61%, a value comparable to those of Anopheline mosquitoes with larger genome sizes (≈ 220 Mb). However, the direct correlation between TE content and genome size has outliers. For example, although *A. quadriannulatus* and *A. gambiae* have genomes with similar sizes, the TE content of the former is less than half of that of the latter (Neafsey *et al.*, 2015, see also Table S1). Although there is variation, in Anopheline mosquitoes, Class I TEs are generally more abundant then those of Class II, as observed also in the present analysis for *An. darlingi* (Table S2).

The increased *A. darlingi* TE content reported in this study, compared to the previous description (Marinotti *et al.*, 2013; Neafsey *et al.*, 2015) is the result of an approach that utilized improved tools and databases. The database used for homology searches in the present study is larger than the one applied in the previous analysis. Also, it is due to the inclusion, in the present report, of short fragments derived from degenerated TEs. The overall proportional representations of the different TE superfamilies in *A. darlingi* were maintained between the present and previous analyses (Marinotti *et al.*, 2013), with *gypsy* as the most abundant LTR element and *mariner* elements as the most abundant ones among the DNA transposons. However, some differences were seen for other superfamilies. For example, *copia* was the second most abundant superfamily observed in this study, but only 0.9% of *copia* elements were registered in the Marinotti *et al.* (2013) study.

Active or mobilizable elements are associated with evolvability of species and their capacity to environmental adaptation (Fablet and Vieira, 2011; Casacuberta and González, 2013). The TE landscape of *A. darlingi* is predominantly formed by degenerated elements, contrasting with species such as *A. gambiae* and *D. melanogaster*, which harbor a larger number of potentially active elements. In *Drosophila melanogaster,* 80% of spontaneous mutations are promoted by TE mobilizations, making it an important source of genetic variability (García Guerreiro, 2012). In contrast, only four putatively active elements were found in the assembled *A. darlingi* genome. It is also remarkable that the copy number found for putative active retrotransposon is very low; only one copy of each element has been identified. Only the putative active *mariner* element (*Mariner11-Andl*) has a higher copy number (12 copies). Judging from this landscape structure, the genome of *A. darlingi* is likely to be stable. Few TEs are capable of, or prone to respond to environmental stressors and likely to promote an increase in mutability.

As a recommendation for future uses of transposable elements as vectors for genetic transformation of *A. darlingi*, attention should be given to the use of *mariner* elements, as it was the only putatively active DNA TE found in the sequenced genome. *piggyBac* elements also deserve certain attention, because a truncated element is present in the genome. The use of other Class II transposable elements is suggested as being safer, as functional elements are not present to promote cross mobilization.

In conclusion, the mobilome of *A. darlingi* is primarily occupied by degenerated elements, showing a minute number of active elements with small copy number, characteristic of a genome that is rather stable.

## Supplementary material

The following online material is available for this article:

Table S1 Comparison of genome size and TE contents in *Anopheles* genus.

Table S2 Percentage of genome occupied by main TEs superfamilies in *Anopheles* genus.

List S1 Description of 36 Identifiable TEs found in the *Anopheles darlingi* genome.
