# Supplementary material for: A re-annotation of the Anopheles darlingi mobilome
Source: Genet Mol Biol. 2019 Jan 21;42(1):125–31. doi: 10.1590/1678-4685-GMB-2017-0300 (PMC6428116; doi:10.1590/1678-4685-GMB-2017-0300)
Supplement: Supplementary file 1 [file 1415-4757-GMB-1678-4685-GMB-2017-0300-s003.pdf]

Supplementary Material “A re-annotation of the Anopheles darlingi mobilome”

List S1 - Description of 36 “full length” transposable elements found in the *Anopheles darlingi* genome.

The “full length” elements or elements large enough to deserve a detailed analysis were separated; 36 are described below.

Abbreviations

bp – base pairs

Kb – kilo base pairs (1000 nucleotides)

TIR – Terminal inverted repeat

TSD – Target sequence duplication

ORF – Open Reading frame

1 – Mariner1-Andl

Degenerated element

Short description

A total of 190 copies of this element were found in the *An. darling* genome, the larger showing 907 bp. TIRs were not found. TSD containing TA sequence were observed. The ORF finder found 8 ORFs, indicating this element is not able to produce active enzymes. Only Orf 8 has a significant hit with transposase.

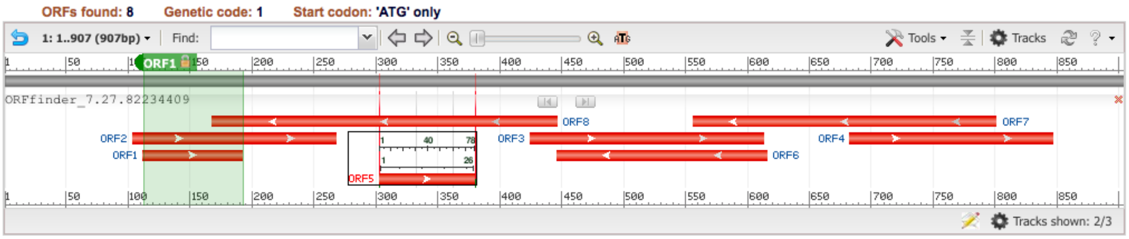

Figure 1- Orf Finder scheme for ORFs detected in the ***Mariner1-Andl*** element

Orf 8 92 aa (Blast hit: putative DD34D transposase [Bactrocera tryoni]/ Histone-lysine N-methyltransferase SETMAR)

>lcl|ORF8
MILAYQTYPPITFGRMFFISSASALDHDNAPSHTSLVFCDFFANIFTHIV
PIKPYAPDLVTFGFWLLDKPKISFRGHCFDTIEQIQAAAKRC

>1 Mariner1-Andl (sequence)
CTACTGTGTCCAAAAATAAGGTGACATTAGATTTATTTTGAAAATTCCTTAATT
TTTCTTCCAAATCATTTTTTTGCCATCAAAGTAATCCCTCCCAGACACAATGC
ACTTATGCCGACGAATTTTCCAGTTTTCGAAACACGTGAAAATGTCTTTAGGA
ATGGCCTTCAGCACCTTTTCGCAGCGGCTTGAATCTGCTCTATCGTGTCAAAA
CAGTGTCCCCGGAACGAAATTTTGGGCTTGTCTAATAGCCAGAAGCCAAATG
TAACCAAATCAGGCGCATACGGTTTTATTGGAACGATATGAGTGAAAATATTG
GCAAAAAAGTCACAGAAAACCAACGAAGTGTGAGATGGTGCATTATCGTGA
TCGAGCGCTGACGCGCTTGAGATGAAAAACATCCTTCCAAATGTAATTGGCG
GATATGTTTGATATGCCAATATCATCAGCAAAGTCTCTTGTTGTTAATCGACGG
TTTTCCAACACCAAATCTTTGAGTTTTTGGCGTGAGCTGCATCGATTGACGTT
GGTGGTCGTCCAGGGCGTTCTTCCTCTTCAAAACGTTCCCGACCCTCCTGGA
AGTCTTTGCACCACCTGTAAATATTTTTTTTGACATAGTATTGTCACCAAAGAC
TTTCTGTACATTGGCATTGGTGTATCCGCAGCAGAAAAAATATTTTTAATGCA
AATTCTTTGCTTAAAAACTTGCGACATGGCGAAAAACGAAGAATGCACTTTT
AGCAGTTCACAAAAAAATACGTATCTCAAACACTAGTGAATATTTTGACGTGA

AACTTGTCATGGTTGTCAATAACAGTGCTGACAACCTACAAAAAATACAAAA
TTAAAGATATCCCTTTACGCGGGCAATTTGGACACCGTGTCACCTTACTTTTT
GGACACAGTAG

2 Mariner2-Andl

Degenerated element

Short description

A total of 32 copies of this element were found in the *An. darling* genome, the larger showing 941 bp. TIRs were not found but TSD consisting TA nucleotide were observed. The ORF finder found 7 short ORFs, indicating this element is not able to produce active enzymes.

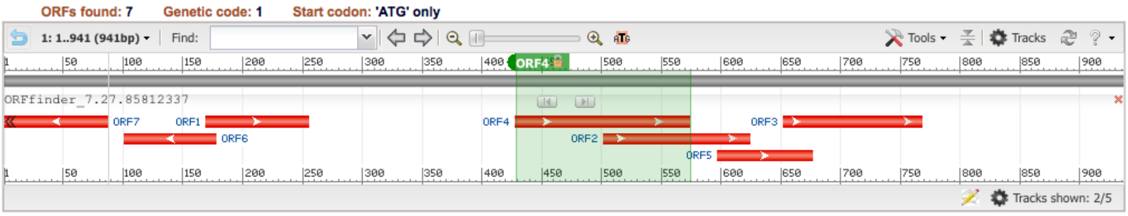

Figure 2 - Orf Finder scheme for ORFs detected in the ***Mariner2-Andl*** element

> Mariner2-Andl (sequence)
CACCGTGTCCAATATATTCTCATACAAATTTTTGCTGAACGTTTTGCTGGATTT
TTGTATATTTACAGCACCTAGCTCAAGTATCATATTTTCCTGTAAACTAGTGTTT
ACTTAGTATTTTGAGAGTTGGATTGGTCTGTTTTCTGTTGGTGGTCGATTTTAG
TAACGATGAAATCATTACGTGCCTGTGTTTTAAAAATCTATCTCGACACGCTG
ACGAGACTTTGTGTTTTTAGTGAAAACTATTTGTTCTAAGCAAGTCATAATGT
CCAAAACGATAGATTGTGGCCAGAAACATCAAGGATGTAACAGATAATCAAA
GAACATCCCATTTTCAAAATGTGGCTAGTATGAAGGGTAGGGTGGTATTTGCA
AGCGCGACAAGTTACCCCTAATTTTTATTGAAAAAGGCATAAAAATTAACTCA
ATGTATTATAAAACAGAAGTTTTAGAGAAAGTGGTGAAACCTACAATTGGAA
AGATTGTACGGGGACGACGATTATGTGCGTTCCAGCAAGATGGAGCGCCCTC
GCACACAGCGAATTTGTCCAAGACTGGTGCAGGAACAATTTAACCAGATTTG
AAGCGAAGAATGAATGGCTCCCAGTTCACCAGAAATCTTAACCCACTGGACT

TTTTCGTTGGTCGTACATGTTATCAATACTAAACGACCATAAACTGTCCAGTTT
GATACAATTCAAAGCAACGATAAATAAAATTTGGATGAAATGCCTATGGAAGT
CGTCCGTGCCCGTGCGATGGGTTTGAGAAGCGATTGAAGCTAGTAAAAAAA
AGTTGGGGGCGAGTAATTTCGAAACATATTTTATAATTAATTAATATAAGTTTC
TTAATAAAAAAAACGCTCGGATTAAAAAAAATGGCCCAATCCTTTAAGAAAA
TATAGATGATTGAAGTTGTATGAGAATATATTGGACACGGTG

3 – Mariner3-Andl

Degenerated element

Short description

A total of 28 copies of this element were found in the *An. darling* genome, the larger showing 1265 bp. TIRs were not found but TSD consisting TA nucleotide were observed. The ORF finder found 14 short ORFs, indicating this element is not able to produce active enzymes.

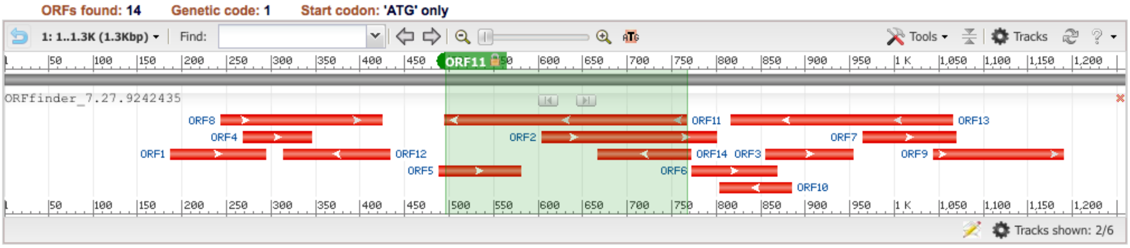

Figure 3 - Orf Finder scheme for ORFs detected in the ***Mariner3-Andl*** element

> Mariner3-Andl (sequence)

TTGGGTCGTCGAAAAAGTCTTTTCGTATTTTGGCAATAGGTGTCTTTGAAGTC
GTCTACCTCCACCGTGTTTATCACATTATCTCATAAATACGGGGGCGTTTTGGG
TGACATTTCAAGTGTCATTTAGTCAAAACAAAACTAAATTTGGAGAAGTTTC
AAAAAAGTTATAGCTGTTCAAAAATGTATGAAAATAATGAAAAATTCGCTAGA
TTTTGAAATTATTTTTAAAAAAAGGGAAGAATGCCCGCAAGCCACCAATCAA
TTTTATGTTTACGGGGACGATGCTCTATAGTTGGTATAGCACAAATGGCTCACT
CGCCTCCGTTCTGGAAATTTCGATGTGAAAGATGCACTCCATCTGGTCGACCT

ATCGTTGCAAAAGTTGATGAAATTTTAGAAAAGATTGACCAGACCGTCACAT
AAGCAGTCATGACATAGCCAAGGAACTAAACATTCATCATAAACCGGCTTTG
GTCCATTTAAAAATGCTGGCTACAAAAAGAAGCTCGATGTTGGATACACATG
AATTGTCTGGAAAATTTTATTGGACTGAATTAACATCTGCGATTGTTTACTGAA
ACGAAATGAAATCGAACCATTCATGAAGCAAATGGTAACAGGAGTCGAAAA
GTGGATCAAATACGACAATAATGTGCGAAAAAGATCATGGTGCAAAAAAGGT
GAAGCTAAACAAACGGCCCCAAAGCCAGGATTGATGCCGAAAAAGGTTATG
CTGTGTGTTTGGTGGGATTGGAAAGGAATCATCCATTATGAGCTCTCAGCCTG
GCCAAACGATTGAATCTACTCTTTACTGTCAACAACTGATGAGATTGAGGCA
AGCAATCGAGAAAAAATGCCAGAACTGATCAACAGAGAGGGCATCGTCTTG
CATCAGGACAACGCTAAACCTCACACATCTTTGATGACTCGCCAGAACTTGG
AGAACTTGTAGGGAAGTTTTGATGCATCCACCATATAGCCCTGACCTTGCACT
ATCGGAAAACCACTTATTTTGTTTGATCAAAAATCCCTTGATGAGTAATGCTG
GCTTCAAGAGAAGCATGTAAAAAATACGTTTCAAAGTTTTTCACCGATAACT
CACGGAATTTTTACACGAAAGGAATAATGTCTTCAGCCGAAAAATGGCAAAA
GATGGCTAACCAACATGGAAAATATTTGGTTGAATAAAGTTATTTAGAAATAA
TGAAAAAAAATCATTTGAAGTTTGATCAGAAATACGAAAAGACTTTTTCGAC
CACCCAA

4 – Mariner4-AndI

Truncated element

Short description

A total of 44 copies of this element were found in the *An. darling* genome, the larger showing 1194 bp. TIRs were not found. However, TSDs consisting of TA were observed. The ORF finder found 9 ORFs.

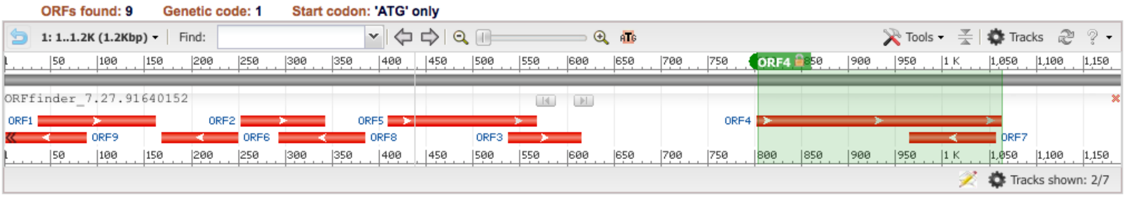

Figure 4 - Orf Finder scheme of ORFs detected in the Mariner4_Andl element

ORF 2

(Mariner Mos1 transposase - Acromyrmex echinatior)

>lcl|ORF2

MLVEAYGDSVLAEITCRIVFVGSKMAILT

ORF 4

(Blast Histone-lysine N-methyltransferase SETMAR, partial Harpegnathos saltator)

>lcl|ORF4

MTKHEKLIILQRQRPSHSSKMVQNYFDTLKGRFYLTPLVLQTWLLQTITC

FRRWVSVADSTSIRMKMPENGLTSGLPHKMKNSLAW

>4 Mariner-2_ACe

CCAGGTGTATAACCTTAAACTGCCGTTTGCTTCTAGATGGGTTTATCGAGCAA

ATTGAGCTGTATCGACTGTCATTTTTTTTTGCATGGGTTCGACACCTGTTACG

TAAATCAGTGCACATCATAAGTGATTTCATGCAAAAACAATTTTTCCTTGCATA

ATCATGTCTAGTTTTGTGCTAGAAAAAGAGCATATCGGGAAGCTCTACTTTTT

TGCTTTAATTTGAAGAAATCGGCTGCCGAATCCATAAAATGCTTGTAGAGGCC

TATGGTGACAGTGTACTAGCGGAAATTACTTGCAGAATTGTTTTCGTCGGTTC

AAAAATGGCCATTTTGACTTGAGTGACAAAAGCGTGAAAATCGACCCAGAA

AAGTTGAGGATCATGAATTGCAGGCTCTTTTGGATAGGATGATGCCCAATCGC

GAAAAATACTTGCCCAGCAGTTACGAGTTACTAAACCAGCCGTTTCCAAACA

TTACGGGCCATGGAATGGTTAAAAATTGGAAAATGGGTAACCATGAATTGAA

CGATATGAAATGGACCCAAAACAGCGAAATTTTGTTTTAGACAAAAAGAATG

TTTTCTGGTACTGCGATGAAGTGGATATATTTTGAGAATCCAAAAAAAAACTG

ATCCCGGAACCACACTTCTTCCAAACAAATCGCTTTGGACGCACGATGCGGC

TTTGTGGGATAGCGGTGTGGCTACTATAGTTTAAACCTGAAGAAACTGTTGCA

TGAGCATCGCTAGCATTAACGACTAATCAAATTGCACCTGCTTTGCATTAAAA

AAGGCCAGTTATGACAAAACATGAGAAGTTGATAATTCTCCAACGACAACGC
CCATCGCACTCGTCAAAAATGGTCCAAAATTACTTTGATACACTCAAGGGGA
GGTTCTACCTCACCCCGCTAGTTCTCCAGACCTGGCTCCTTCAGACTATCACC
TGTTTTCGCCGATGGGTCAGCGTTGCCGACAGCACTTCGATTCGTATGAAAAT
GCCTGAAAATGGCTTGACGAGTGGTTTGCCTCACAAAATGAAGAATTCTTTG
GCATGGTAGACACAAAGTGCCCAAAAGATGGGAAAATGTGTAGCTAGCGAG
GGAAAATATTGGATAAATTAATTTGGCTTTCTATTTAAAAAAATGTGTTTTTT
CGACAAAAAAAAGCAGAGTTAAGGTTATACACCTGG

5 – Mariner5-Andl

Degenerated element

Short description

A total of 91 copies of this element were found in *An. darling* genome, the larger showing 890 bp. TIR were not found but TSD consisting TA were observed. The ORF finder found 11 short ORFs, indicating this element is not able to produce active enzymes.

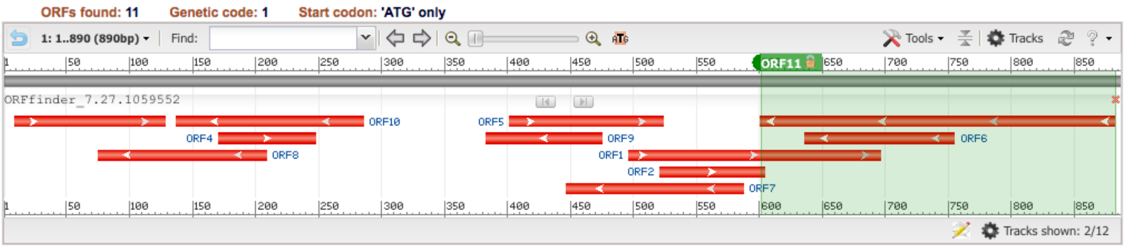

Figure 5 - Orf Finder scheme of ORFs detected in the Mariner5_Andl element

> Mariner-5_Andl (sequence)

CCAGGTGTATGCATATGAAACCGCCCTCCGTGTTTATAGCTATATCTATGCCAA
ATGGAACCGTAGAACCGTTCTCAATGTTTCTTTTTGTTCGTTCGGCATCTGTT
AATCAAAATCGTGCGCAATAGTTCTAATTTCACACTACAACCAATATTTTCATT
GTGTACAAAATGGCAAGTTCCGTCCTTAAAACTGCAAAATGCGGACATCGTT
CATTTTCTGCCTCTATTTTCAAAAATAGGCGATAGAATCGCATCAAATGCTTAT
CGAAGCTTACGGTTAGCATGCTCTTTATGAAACACAGTGCAAAGAGTGGTTT
TAAAAATTTAAAAATGGCGATTTTGATGTGACAAACGAGAATCGCGAGAAAC
CACCGAAAACGTTAGAAGACACCGAATTGCATGCCGTATTGGGCGAAACGGT
TATTAGGAGCTATACAAGCAACACTATCGATTTCGGACCGTGAAATCCGCATA

AATCGACCGGAATATCGAAATGATAAACAGACCGATTTTGCTCGATGAAAATG
CTCGCCACACCGCGGAAAACCGGCCAAGAAGACTATACAAGACCTCAATTG
GGATCATTTATCGCTTGCGGCTAACTCACCAGACTTGGCTCTATCGAATCAAC
ACTCATTTGCATCGTTGGGACAGATACTGGCCGAGCAGCGCTTCCATTCTTAC
AAAAGTTTGAAAAAAATCGACCTGTGATTGGTTTGATACAAAAGACCGACTT
GTTTTTTTGCGAAGCATCTGTAAATTGCCTAAAAGATAGGAAAAATGTATAGC
TAACTATTGCCATTTCTTTGAAGAATAAGAGTTTCTCCATTTTCGCAGAATAAA
AATTTTTTTCTGAAAAAAAAGGCGGTTTCATATGCATACACCTGG

6 – Mariner6-Andl

Degenerated element

Short description

A total of 30 copies of this element were found in *An. darling* genome, the larger showing 905 bp. TIR were not found by TSD consisting TA were observed. The ORF finder found 9 short ORFs, indicating this element is not able to produce active enzymes.

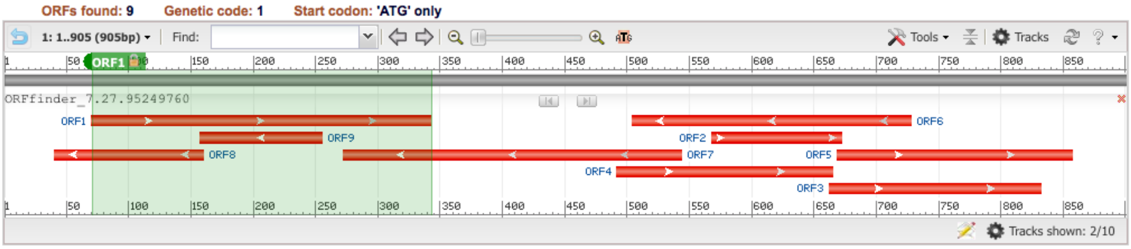

Figure 6 - Orf Finder scheme of ORFs detected in the Mariner6_Andl element

ORF1

(Blast Mariner Mos1 transposase -Acromyrmex echinatior)

>lcl|ORF1

MQNPGNYSWGICQQLPKISFVYFHSALSYSCANMAKFEPNKCHLREVLLF
AFHTKKNAAEAHRMIVKFMVKPALVKEVSGVVLQVQKRRL

ORF 4

(Blast - Mariner Mos1 transposase -Hymenolepis microstoma)

>lcl|ORF4

MIQKLGYWVPHELKPRDIERLRRRNHENIPGNIKMAHYPTRCTLQTSLHQ
TTIYSDR

ORF5

(Blast - Mariner Mos1 transposase - Hymenolepis microstoma)

>lcl|ORF5

MTWPSSASVLSRTPKIGSMHGSRRKMNNFSHEEFKSCPNCGKKLWLTMAI
TLKNEFVRGFLQ

> Mariner6_ Andl (sequence)

TTAGGTGTACAAATAGAAACCGCCGTTTTCCCATAGGGGTCTAGCAGCTGCA
AATTAATCGATAAAAATATGCAAAACCCTGGTAATTACAGTTGGGGCATCTGT
CAACAACTCCCCAAAATCTCGTTTGTTTATTTTCACTCTGCTTTGAGTTATTCA
TGTGCGAACATGGCGAAGTTTGAGCCGAATAAGTGCCATTTGCGGGAAGTGT
TGCTTTTTGCGTTTCATACTAAAAAAAATGCAGCTGAAGCGCATCGAATGATA
GTAAAGTTTATGGTGAAGCCTGCATTAGTGAAAGAAGTGTCGGGAGTGGTTT
TGCAAGTTCAAAAACGGCGATTATGACGTGCCAGACAAGGAGCGTCCCACA
AAAAGTTCGAAAATGCTGAATTGAACACTTTTTTGGAAGAAGATTCTTGTCA
AACGCAACAGGAACTTGCAGACTTATTGTGCGTGACGTAACAAGCAATATCG
CATCGGCTAAAGCCCTGGGAATGATCCAGAAACTAGGATACTGGGTGCCGCA
CGAGTTGAAGCCGAGGGACATCGAACGGCTTCGACGGCGGAATCATGAAAA
CATACCTGGAAACATTAAAATGGCACATTACCCCACCCGCTGTACTCTCCAGA
CATCGCTCCATCAGACTACTATCTATTCCGATCGATGACGCATGACCTGGCCG
AGCAGCGCTTCCGTTCTTTCGAGGACACCCAAAATTGGATCGATGCATGGAT
CGCGTCGAAAGATGAACAATTTTTCCCACGAGGAATTCAAAAGCTGCCCAAA
TTGTGGGAAAAAGTTGTGGCTAACTATGGCAATTACTTTGAAGAATGAGTTT
GTAAGGGGTTTTTTACAATAAAGCCTCAAATCATGAGAAAAAACGGCGGTTT
TCTATTTGTACACCTGA

7 – Mariner7-Andl

Degenerated element

Short description

A total of 99 copies of this element were found in *An. darling* genome, the larger showing 912 bp. TIR were not found but TSD consisting TA were observed. The ORF finder found 9 short ORFs, indicating this element is not able to produce active enzymes.

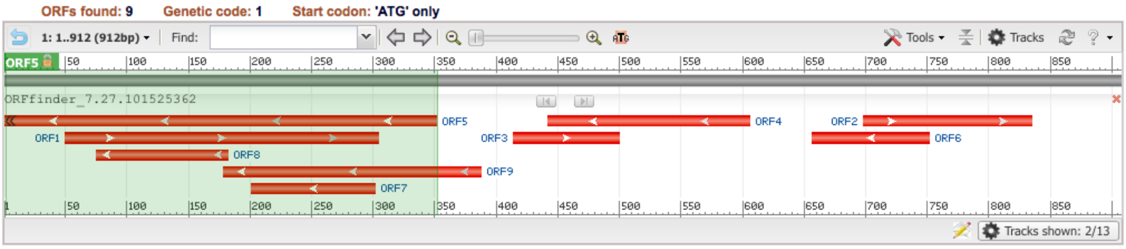

Figure 7 - Orf Finder scheme of ORFs detected in the Mariner7_Andl element

ORF1

(Blast - transposase -Echinococcus granulosus)

>lcl|ORF1

MKIERSRFFSCLVGLSGIGKAPWIASLTLSVSTVIILLHEVPSSFAQHII

IKFLAVEGVKLIEILRRSTAYFREETLSRARVVA

> Mariner7_ Andl (sequence)

CGAGGGTGAATCAAATATAAACGAGACTTTTCGTCCTACGGTCACAAAAATG

AAGATAGAACGTTCTCGCTTTTTTAGTTGTTTGGTTGGTTTGTCAGGAATAGG

AAAAGCGCCCTGGATCGCCTCATTGACACTCTCAGTGTCCACAGTAATAATTT

TGCTGCATGAGGTACCTTCGTCATTTGCGCAACATATTATAATCAAGTTTCTCG

CCGTAGAAGGCGTAAAACTGATCGAAATCTTGAGGAGATCGACTGCATACTT

CCGGGAAGAGACACTGTCGCGAGCCAGAGTGGTTGCATAGCTTAAAAAAAT

CTTTTTGGGTGTAATCGTGTTGAAAATGAGAGCCATAACCGCAGACCTCGCA

CCAGCCTAAGAGCGCAAACATTAAATCCTTAGACGGCTTACTGAGAATGACA

GAGTCGTATCACTACAGGACAATTAATGCGACCGATTCTTGTAATGTTTTAAG

TGAGATCCGACTTGCATATCGCCTCTAAATATGGGACATATTGATAAGGGAAG

TGGTTCTTCTTCATGACAATGCTCGCCCACAACACTTGCAGCCATTACTAGGG

AAAAAAAAGCAACTGAGGTGGGAAACATTGGAAACCCTCGTTACAGTCCTG

ACCTGTTACCCTGTGACTTTCATGTGTTTACTCTCTCAAGGAAATTTTGGGAG
ACAAAAGATTCAACAATGATACTGAGGATGAAACCTCATTTGCGCAATTGGC
TGCAAACACGACCAACATCTTTCTACGAGAAATATATTAAAAAGCTGCCTATC
CGCTGGGAAAAATGTGTTTCGGAATCAGGAGATTATGTAGAAAAATAAAAAA
ATACCTTTTGTGCGTTTAAACTAAAAATAAATTTGCTAAAAATAAAGTCTCGT
TTATATTTGATTCACCCTCG

8 – Mariner8-Andl

Truncated element

Short description

A total of 20 copies of this element were found in *An. darling* genome, the larger showing 1675 bp. TIR were not found by TSD consisting TA were observed. The ORF finder found 8 ORFs, indicating this element is not able to produce active enzymes. However, two large ORFs (148 and 141 aa) showed hits with transposase, indicating this element became degenerated recently.

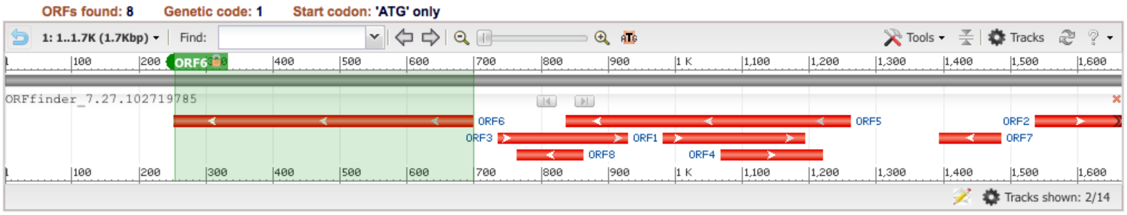

Figure 8 - Orf Finder scheme of ORFs detected in the Mariner8_Andl element

OFR6

(Blast -Transposable element Tcb1 transposase, partial - Camponotus floridanus)

>lcl|ORF6
MGWGSFSWAGVGNLVHIHGIMTSDVYIDILKGNLTNSARRAGLRRHFILQ
QDNDPKHTAKKTTAFLRSRSVKMLEWPSQSPDLNPIENLWALLDRKVVKD
NVTSKESYIHALQEAWATIEPTHLQNLVNSMPKRLQMVIEAKGSHINY

ORF 5

(Blast - transposable element tcb1 transposase -Lasius niger)

>lcl|ORF5

MGGKKISLQVRNLVLKDNFNNVSQRKIAEKFSISRGAVRAIIAKHRIFGS
VNDRSKTGRPRKTDHRMDVRIIREIKKSPKTTIRSIKESLSLSISDRTIR
RRLVQHGYESRLAKRRPFISKKNKLKRLEFARKYVSKTKEF

> Mariner8_ Andl (sequence)

CAGTGACCGGCATAAGTAAAAGCCCACCCCTAGTTTTTGTACATTTTCGCCT
GTAAATCCAACAATTTTGGTCGCAAAAGGCAAGTGTTATGTTCTTATAGAAGG
TTTAACATTCCTCTTTCAGATTCACTTGCTTTCATCTCGATTGGATTTATAACTT
TTTTTTGAAAATTCTTTGAGTGAGAGATGGTCGAGCATGATTATGACAACAGT
AAAAGCCCATACGTATTAATATGTTATTATTTATTTATTAATAGTTAATATGGCTT
CCCTTAGCCTCAATCACCATCTGCAAACGTTTTGGCATGCTGTTTACAAGATT
TTGTAGATGTGTTGGCTCGATTGTTGCCCATGCTTCTTGTAGAGCATGAATATA
TGATTCTTTACTCGTTACATTATCCTTAACTACTTTTCTATCTAACAACGCCCAT
AAATTTTCAATTGGATTTAAATCAGGGCTTTGAGAAGGCCATTCTAACATCTT
TACCGAGCGAGAACGTAAAAAAGCGGTGGTTTTTTTGGCAGTGTGCTTCGGA
TCATTGTCCTGCTGCAAAATGAAATGTCTGCGTAATCCGGCCCTTCTAGCTGA
GTTAGTCAGGTTGCCTTTCAAAATATCAATATAGACATCGGACGTCATAATACC
GTGTATATGGACTAAATTACCCACACCAGCCCACGAAAAACTGCCCCACCCC
ATAACACTACCGCCGCCGTGCTTTACTGTGGCCTGCAAATGCCGCTCTTTCAA
AGCCTCATTTGTTTTCAACCAAACTCTTTCGCGCCGTTTGCTATTAAATATCTC
GAATTTAGATTCGTCGGACCACAAAACACTTCTTCAAAATTCCTTTGTCTTAC
TAACATATTTTCGGGCGAATTCCAATCGCTTGAGCTTATTTTTTTTACTAATGA
AAGGACGTCTTTTAGCTAATCTACTTTCGTATCCGTGTTGAACTAACCTACGT
CGTATAGTACGGTCCGAAATGGACAAGGAGAGAGATTCTTTGATCGAACGGA
TCGTTGTTTTAGGGCTCTTCTTTATCTCACGAATGATTCTTACGTCCATCCGAT
GGTCCGTCTTCCTAGGGCGTCCTGTTTTCGATCGGTCGTTAACCGATCCAAAA
ATTCGGTGTTTTGCTATAATAGCCCTAACTGCTCCCCTACTTATAGAAAATTTT
TCTGCAATCTTTCGTTGAGAAACGTTATTAAAATTATCTTTTAGAACAAGATTT
CTCACTTGCAAAGAAATTTTTTTCCCTCCCATGCTATTTTTAATTGCAAAATCT

ACTGCGCAATCAACTCTGTGCTAGCTATACTACGGCTGGTAGACTAAATCAAA
GTAATCAAATATCAAGGTCATCTAAAACGTCAAACTAGATGTAAACGAGGGG
CCTATCAAAGATATACTATCACAAGGGGCTTATCAGAGGCACAATATTGTTTTT
TGCAAAGTGGGCTTTTACTTTTGTCATAATCATGCTCGACCATCTCTCACTCA
AAGAATTTTCAAAAAAAAGTTATCGATCCAATCGAGACGAATGCTAGTGAAA
CTGAAAGAGGAATGTTCAAGCTTTAAGAAAACCACTGCACTTGCCGGTTACG
ATAGAAAAGGTAGGATTTATAGTCAAAAATCTACAAAAATCAAGGGGTGGGC
TTTTACTTATGCCGGTCACTG

9 – Mariner9-Andl

MITE

Short description

A total of 10 copies of this element were found in *An. darling* genome, the larger showing 738 bp. The TIRs have 223 pb and TSD consisting TA. The ORF finder found 9 ORFs, indicating this element is not able to produce active enzymes. These characteristics suggest that such sequences are MITEs.

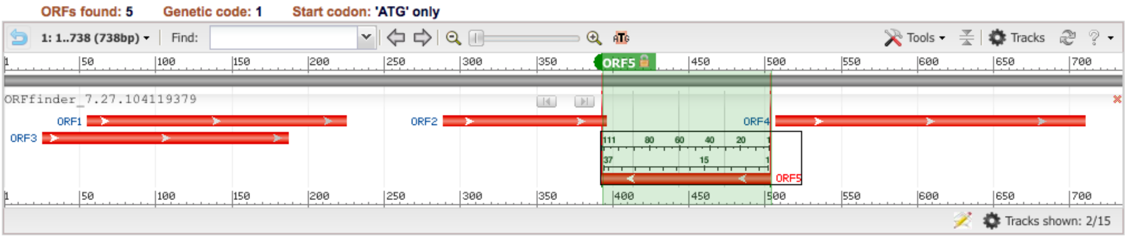

Figure 9 - Orf Finder scheme of ORFs detected in the Mariner9_Andl element

ORF 5

(Blast -transposase -Ceratitis rosa)

>lcl|ORF5

MIKIFLCPEIDGMNLDEMLFHQNGATCHTSDETIFI

> Mariner9_ Andl (sequence) Red = TIRs

AAGGGTGGTCCAACTTAGGTGCACGATGTTGATTGGTTATAAAACACACAGT
TTATGGAAAATACCGTTATTTGTATTTTTTTTGTTAGATCAATGCTTGACAATTA
TGTGTGGAACAATATATCGCCCATATGTTCACCACGGCTTTTGTGGCAAACCG
CTATTCGTTGGTCCATATTTGCGATAGCTCGGCTACATAAAATTGGATCTATGT
CCCTGGTTTGACGAATAATCTCGTTTTTAAGCTCTTGTATGGTGCGTGGTTTAT
AAACATACACTTTTTCTTTAATGTATCCCCAAAGAAAAAAATCCTGGGGAGTT
AAATCACAAGATCGGGGCGGTCAATTCACGTCGCCACAACGAGAAATTATCC
GGTCTTTGATTTTGACGCTTTAAATGAAAATGGTTTCATCGCTTGTGTGGCAG
GTGGCGCCATTTTGGTGAAACAACATTTCGTCTAGGTTCATACCATCGATTTC
AGGGCACAAGAAGATTTTTATCATCTCATGATAGCGAACACCATTCACAGTAA
CCGCTTGGCAACCGTATCTTCGAAGAAGTACGGCCCGACTTTAACGCGGTGT
TTCATCACTTTAACGCGCTGTTACAACGAATATCGCTGCATGATTGAAATTGTC
AAACATCCGACTAGTAAAATATCAAAACAAATCGTGTCTAAATCGAACGATTA
TCGAATGGTAGACATTTAACATCGTGCACTCAAGTTGGACCACCCTT

10 – Mariner10-Andl

MITE

Short description

A total of 2 copies of this element were found in *An. darling* genome, the larger showing 489 bp. The TIRs have 22 pb and TSD consisting TA. The ORF finder found only 1 ORFs. However, this ORF is very short (22 aa) indicating this element is not able to produce active enzymes. These characteristics suggest that such sequences are MITEs.

ORF 1 -no significant hit

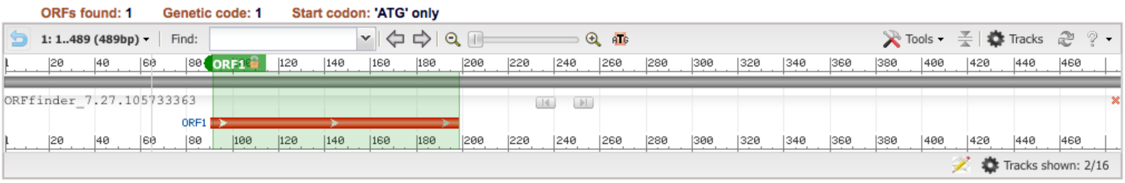

Figure 10 - Orf Finder scheme of ORFs detected in the Mariner10_Andl element

> Mariner10_ Andl (sequence) Red = TIRs

TATCGTAAAAAAACCAAATTAAGTCCGATTTTGCACTCAGCGGTATATGGTGG
GTGCATAAAAAGTTCATAACCAAGCTCTCTAAATTGAATGCGATGCAAAGTTG
AATGCGTCCTGCTTTGCTCTGGAGGACTATATTTTTATGATTTCTGTCAGCTTC
TCGTTAAGGGTCCATCTAATTTGATCAATTTTTGGTAGTAGACATTCAAATCAA
TCATTTGATTCCTTGGAAGCGTCTCAAAAGTTACTATACTCTTCCAACTCCAC
CAAACTGACAACAATAATCTTGTTTTGGTGAATACAAATTTTTGAAGTCCTGT
TGAACTGGTCCTCAAATTTAATCCGTGATCATTTTTGACTTTAGTTACTGTAAA
CAATTGAATGTTGGTTAATAATGTTGTTTTGTCCGAAAATGAATCAAATTCATT
ACGTTAAGCAAGAAATACATAAGGGTTGAAAAAGGTTTGTTAAATTGAATTT
ATTCCGATA

11 – Mariner11-Andl

Potentially active element (at least recently)

Short description

A total of 12 copies of this element were found in *An. darling* genome, the larger showing 1285 bp. The element has TIR 230pb long and TSD consisting TA nucleotides. The ORF finder found 12 ORFs. However, two larger ORFs (ORF 3 and 1) codify a transposase with 301 aa, compatible with an active transposase. The assembled copies showed a frameshift, but it is possible there are functional copies in the populations.

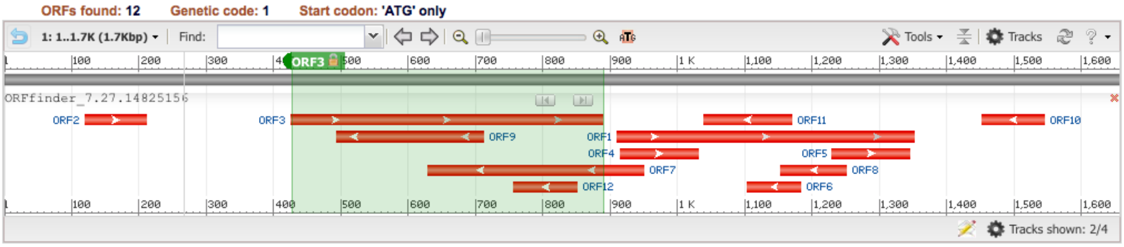

Figure 11 - Orf Finder scheme of ORFs detected in the Mariner11_Andl element

ORF 3

(Blast - transposable element tcb1 transposase - Lasius niger)

>lcl|ORF3

MFREVQDIMCCYAKKIANALKWNYTPESRGKKRATSAQTDREIIRTVKKR
PQISSLALKKELNLSISTSTIRKRLREANLYARVPRKIPLLKPRHIQNRI
MFAKSRVNWPLTKWRNILWTDESKVELFGSGGQRKFVRCPPPFWNEPQIY
CKHS

ORF 1

(Blast - transposable element Tcb1 transposase isoform X2 [Drosophila bipectinata)

>lcl|ORF1

MVWGCFSYSGVGPIHRIEGIMNAVGYVDILQNIMLPYAENELPLKWVFQQ
DNDPKHTSKRAKAWFQERKIDVMEWPAQSPDLNPIEHLWTDIKRAVNKAM
PKDSEQLWAAVKAAWYQIPTSRCQNLMDSMPRRCAAVIKHKGHATKY

> Mariner11_ Andl (sequence) Red = TIRs

CAGCATTGGTCGATAAAATAGCAGCGCAAGTGCGATACGCAAACTTGCGCGT
TGTACTCGCTCACGCTTTCTTCTAATTAATTTTTATCGCATCTGTTGGGTGCATT
AGCGTCTGTGGAATGCGGTTTGGTGTTAATTTTAATTATCGATCGCTTAGGTAT
CAATATATAGGTATCTATTGCCAAAACCGTCTGGTTCAGGTTGGTCGATGAAAT
AGCAGCGCTAACGAAGAAGTCTTTTTTTAGTGACGCGAGTTTTTTTTGTGAA
ATCGCAGTTGTACTACTGGGTAATGTGAAGAATTTGCGGGTTTTTACACTAGG
TTTGAAACATCTAACGTAAGTTCAATTTTCAGTAAAATGGGGCGTTCAAAGC
ATTGAACGGTTGAAGACCGAAATGCGATAAAACGATTGATATCGGGAGGAAA
GATGTTTCGGGAAGTGCAGGATATAATGTGCTGCTATGCGAAGAAAATAGCA

AATGCACTGAAATGGAATTACACACCCGAAAGTCGTGGAAAAAGCGCGCA
ACAAGCGCACAAACTGATCGCGAGATAATCCGAACGGTGAAGAAAAGGCCA
CAAATTAGTTCTTTGGCATTGAAAAAAGAACTAAACTTGTCTATCAGCACATC
AACGATAAGGAAACGTTTGCGGGAAGCAAATTTGTACGCCAGAGTGCCGAG
GAAAATACCCTTGCTTAAACCAAGACACATTCAAAATCGAATAATGTTTGCCA
AGTCCCGGGTCAATTGGCCACTTACCAAATGGAGGAATATTCTTTGGACTGAT
GAGAGCAAAGTGGAGTTGTTTGGATCTGGGGGACAGCGAAAGTTTGTGAGA
TGTCCCCCCCCATTCTGGAACGAACCCCAAATTTACTGTAAACACAGTTAAAT
TTGGAGGAGCCAAGGTTATGGTATGGGGCTGTTTCTCATACAGCGGTGTAGG
TCCCATTCACCGTATCGAAGGCATCATGAATGCTGTTGGATATGTCGATATCTT
ACAAAATATCATGCTACCATATGCGGAAAATGAATTGCCGCTAAAATGGGTCT
TCCAACAAGACAATGACCCCAAGCACACCAGCAAACGTGCAAAAGCTTGGT
TTCAAGAAAGAAAAATTGACGTAATGGAATGGCCCGCACAATCCCCCGATCT
CAATCCGATCGAACATTTATGGACGGACATAAAAAGGGCTGTGAATAAAGCA
ATGCCAAAAGATTCTGAGCAGTTATGGGCCGCAGTAAAGGCAGCATGGTACC
AAATACCAACAAGCCGCTGCCAAAACTTAATGGATTCGATGCCACGAAGATG
TGCAGCCGTTATAAAACACAAAGGTCATGCTACTAAATACTGAGCACTTGTCA
ATGTTTTCTAATTGAAATCTAATAAAATTCGAATACAAATTTGGCTTTTTTGTT
AATAAGAACTCGTTGAGTTTGTTAGCGCTGCTATTTCATCGACCAACCTGAAC
CAGACGGTTTGGGCAATAGATACCTATATATTGATACCTAAGCGATCGATAATT
AAAATTAACACCAAACCGCATTCCACAGACGCTAATGCACCCAACAGATGCG
ATAAAAATTAATTAGAAGTAATCTTGAGCGAGTACAACGCGCAAGTTTGCCTA
TCGCACTTGCGCTGCTATTTATCGACCAGTGCTG

12 – Mariner12-Andl

Degenerated element

Short description

A total of 46 copies of this element were found in *An. darling* genome, the larger showing

1285 bp. TIRs are 30bp long and TSD consisting TA nucleotides. The ORF finder found 9 short ORFs, indicating this element is not able to produce active enzymes

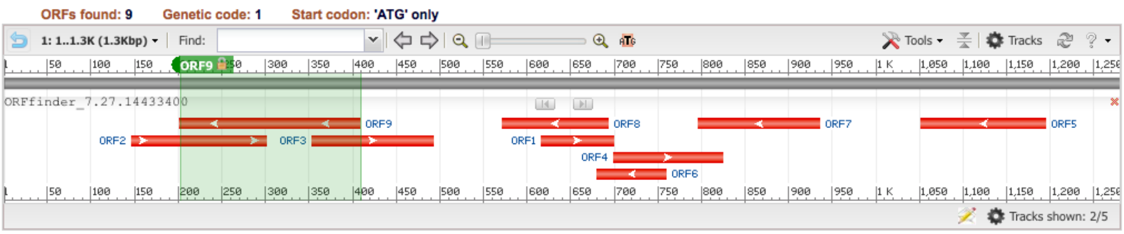

Figure 12 - Orf Finder scheme of ORFs detected in the Mariner12_Andl element

> Mariner12_ Andl (sequence) Red = TIRs

TACAGGGTGGCAACGTGAAAGTGACACACTTATTCCAGGGCCCCCTGTACTA
ATTCGAACAGCTATAATTCAGATCTTGGTTTACAGTTTGAAATAGCAGTATTTT
TACGGCTGGGTTTGAGTGTATGGTCACCCAAAAATTGTAATGGATTCTTGTAG
CGAACAAATCAGATTGTTGTATCTTCAAATCCTCATCAACATTCAAATAAGAG
GAAGGCTGTCACATCTGAATTTGAGCAGATTATGCGCGTACAGAACCATCAA
ACGATTCAAATATAGTGATATGGTTGTCCCCAAATAGAAACCGGTAAAAAAC
GGACTGTACGAACTCTAACGACTATTAATGCGGTGATGGAGCGCATACGACG
AAATCCGGAACGATCGGAAAGAAAAATGGCTAATGGCATGAAAATGAGTCA
CACCTGCATGCAAAACATAATAAAAAATACCTTAGATATAGTCCTTTCAAGAA
GCAGAGGATTCATGGGTTGACTCGCAAAAACCTCGCCGACAAGTTTGCTAGA
TCTCGGATGCTGCTGAAGACGCACGCTGCTCACAACATATTTTTTCAGACAA
CAACAAAAGACTTTGGAGGCAACTTTGAACAAGCAAAATGATTGCGTGCATA
GTGTATCTTTTCGCGGTATTCCAACCGATAAAAATGGTGCGGAGCGATATCAA
AATGGCTCATATGTGATGGTACGGGGCGCCATATTAAAAGAAATAAAACTCCC
ACTGTTATTTGTTGATAAAGGCATAAAAATCCACAAAAAATATTATTTGGAGTT
CGTTATCAAGCTCCATTTGATTCCTTGTGCGTAGAAAATATAAGGAAAAATAG
ATTTTTGTTTCCAACAGGCTTCAACACCTGCGTATAAGGCATTGACTGTTCAG
ACATGGTGAGCGCAAAATTTCCCATGCTTTGAAAGCTCATCGGAATGGCATG
TATCTTCTTCAGATTTAAACGCACTCGATTTCTAAGCGTGGGGATACTTGCTAG
GGAAGCTGGTTGACACAAAGAATATGACTTTAATACGTTAAAAAACGGTTTAT
AAAAATTTGAGACAAAATACCGAAGAGAGACGTCCGTGCGGCTGGCAAAAG
TTTTGGAAAGCGATTAGGGGCTGTAATAAAAAACAAATGCGAAAGAATGGAA

ATGAATCAATCAAATTGAAACAATAGCGATAGGACATTGTGTTAAAAATGAGA
AATACGTGAAAAATCACATAGTTTCCATTTTATGACAAAATTTAAGTGTAGCA
CTTGCACGTTGCCACCCTGTA

13 – Mariner13-Andl

Degenerated element

Short description

A total of 64 copies of this element were found in *An. darling* genome, the larger showing 906 bp. TIRs are 64bp long and TSD consisting TA nucleotides. The ORF finder found 5 short ORFs, indicating this element is not able to produce active enzymes

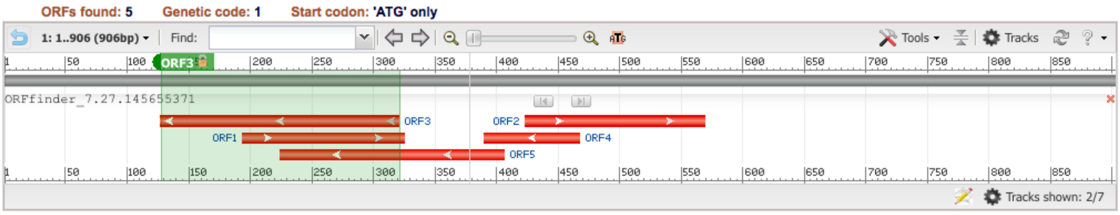

Figure 13 - Orf Finder scheme of ORFs detected in the Mariner13_Andl element

ORF1

(Blast - putative DD34D transposase - Bactrocera tryoni)

>lcl|ORF1
MGDDVKQRICLKFCFANEMSCADALKMLQKAFGNNTPIENSCI

> Mariner13_ Andl (sequence) Red = TIRs
CATTCTGTCAAGAAGTACCGGAATAGTCGATTTAAATCTGACTGGGCTGTCGG
ATCAATTGAAGTCTTTTTTTCCTAGGTTGGTACAACTGTCCTTAAATATTGTGC
AAAATTTGAGCAAAATCCGTCAACTCGTTTGTTTACAGCCATTTTTTAAGTAA
GTCGATGTCAGTAGTGAGCGGCGATTTGTTATCATGGGTGACGACGTTAAAC
AAAGAATTTGCTTAAAGTTTTGTTTTGCAAATGAAATGTCGTGTGCCGATGCA
TTGAAAATGTTGCAAAAGGCCTTTGGCAATAATACTCCAATCGAAAACTCGT
GCATATGAGTGGTACAAAGCCTTCAAAAGTGTTTGGACAGATGCCTTGATTTC
TGATTGTTCATTAACTTCCTCAACCGATGAAAACATCGATAAAGTGAAAGATA

TGGTGTTCGAAAAACATCATTATAGCTTCAGAGAGTTGTTTCATGAACTTAAC
ATATCGGTTCGTAACATTTGATTGATTTTTGGACATGAAACGTGTTGCAGC
AGAACTCGATCCAAAAGAAGTTTTTTTACATTTTTTTAATTTTCTATTTTTCG
CTCTAAAACTAAAAAAATATCATCGAACAACCACCGTATTAACCAGAATTAGT
TCCCTGTGACTTTTTACTTTCCCCAAAACTTAACTTGCCCCTTCACGGCATCT
GTTTTGAGTCGATTTAAGCCATAAAGCAAAAATCGCTGCTTACTACTGACATC
GACTTACTTAAAAAATTGCTGTAACAAACGGGTTGGCGGATTCCGCTCAAA
TTTTGCACAATAGTTAAGGGCAATTGTATCAACCTAGGAAAAAAAGCTTAACT
TGATCCGACAGCCCAGTCAGATTCAAATCGACTATTCCCGGTACTTTCTTGAC
AGAATG

14 – Mariner14-Andl

Degenerated element

Short description

A total of 16 copies of this element were found in *An. darling* genome, the larger showing 1321 bp. TIRs are 33bp long and TSD consisting TA nucleotides. The ORF finder found 17 short ORFs, indicating this element is not able to produce active enzymes

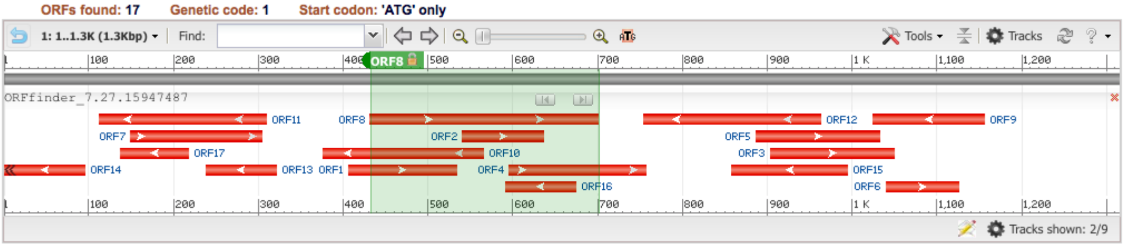

Figure 14 - Orf Finder scheme of ORFs detected in the Mariner14_Andl element

ORF12

(Blast - putative DD37D maT transposase -Bactrocera tryoni)

>lcl|ORF12
MVQKLKKRIQRNPRRSGNQHARDLSISRRSVRRILTHELGLKALKIQKVQ
EVTDAQKKLDSKDRKTCFV

ORF 11

(Blast - putative DD37D maT transposase -Bactrocera tryoni)

>lcl|ORF11
MDYCVWGILEAKVSNKKYSSIDQLKQALRREWAKIPQEQLRTACEGFVGR
LKAIVRAKGGYFEQT

> Mariner14_ Andl (sequence) Red = TIRs
ATACAGGGTGCGGCAAAAAAAAACTGCAACAAAGTAAATCATCATAGTTTTT
TCAATTTTTATTGAATTTAATTCAAAAATTGTTAGAAATTTCATAACATTTTTAA
AACAGTTACGTTTGTTCAAAATAGCCACCTTTAGCACGAACAATGGCCTTTAA
ACGCCCGACAAATCCTTCGCACGCTGTCCGCAGCTGTTCTTGGGGAATTTTG
GCCCATTCTCTGCGAAGGGCTTGCTTCAGCTGGTCGATAGAACTATATTTTTT
GTTGGAAACCTTGGCTTCCAAGATACCCCAGACACAATAATCCATCGGATTAG
CATCAGGGGATCTTTGGGGCCATATTGTGTCGCAGTAATGAAATTCGGAACCT
CTGCTCTAAGCCATTCTTGAGTGGCGCGCCGAATGAGACGGTGCCGAGTC
TTGTTGAAATGTCCAGCGTCGGTGTCCGAAATGTTTGTGTGCCCACGGCTTC
AACACACCCTCCAGGATGTTTTCCCGAAAATATTCGGCATTTATCTTTATGTAG
CATAAAGATAAATGAAAACGAGCGGAGAGCGAACATCAGCAGTTATCCGCGG
CCCACACCATCACCATGGCCGGCGCTTGTTCTCTGGTGGCCAACCGAAGATG
TAAATTATCAGTTGACCTCTTTGGTAAGTCAACACGATCATTTTGTTTGTTTAC
AAACTGCTGGATAAGGAACGGTTTCTCATCGGAGAAAACCAAATTCGGAATT
TCCCCACGCGCGGCCTAGACGAAGCAAGTCTTTCGATCTTTCGAGTCTAACT
TTTTTTGGGCATCTGTTACCTCTTGCACCTTTTGAATTTTTAAGGCCTTCAGTC
CAAGTTCATGTGTCAAAATTCGTCGGACGGAACGCCGCGATATGGATAGGTC
ACGGGCATGCTGGTTGCCGCTGCGCCGTGGATTTCGTTGGATCCGTTTCTTGA
GTTTTTGGACCATTGCTGGTGCAGTTGCAGTTTTTTTGCGGCCATTTCCTGGA
CGCCGGGCTACACTACCAGTCTCACGGTAGCGAGCGATGGTGCGTGACACAA
ACGATTTGTTCACATTCAAATGTTTTGAGGGCGCTGACGATATCAACTTGTGC
TTTTCCACCCAAGTGTAGTGCAATCACACTTTCACGCTTCAGCTCCATGGCGA
TTTTTAGAATTACCGAAAGTGATGCAACAAAATAAATTCGTACGATGGTTTAC
TACTAAATGAACTGTCACTGGAATAATTTTGACAGCTGTCAGTCGAGCAGATT
TGGAATGGCAGCTATTTGAAGTTGGTTGCAGTTTTTTTGCCCTACCCTGTATC
A

15 – Mariner15-Andl

Degenerated element

Short description

A total of 31 copies of this element were found in *An. darling* genome, the larger copies have an estimated size of 1220 bp. TIRs are 23bp long and TSD consisting TA nucleotides. The ORF finder found 9 short ORFs. However, there are long nondeterminate sequences (NNNN) which difficulties a more accurate analysis.

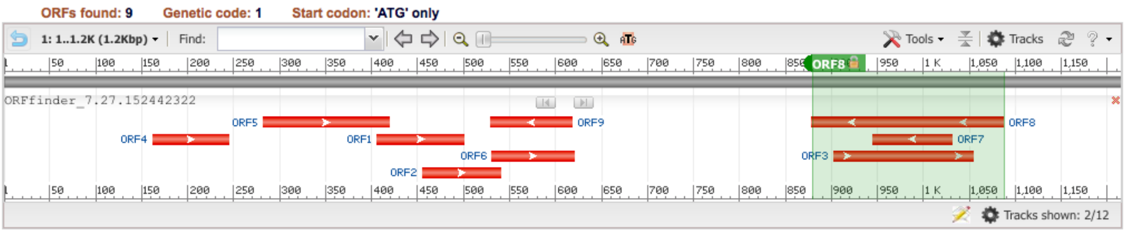

Figure 15 - Orf Finder scheme of ORFs detected in the Mariner15_Andl element

> Mariner15_ Andl (sequence) Red = TIRs

CAGGGTTACTGAGATGAAGTGTTACCAACTTTACACTGCTTGTATCTTTTGAA
CCACTCATAACTTCAACGCCAAAATTGTTCTATTGAAAGTTCAATATATTGTCT
ACAAGCCACTAATAGCGTTTTAATCTCAGTTTTACTGAATATTTTTTTACCGTC
ATGGAATTAAAAATTAAATTTGCATTTGTATATTTGGCAAATCATAACCAGCTA
CTGTTCGTGAGCTCAATCACCTTAAAGTGAATGAAATATTTGTGTATCACACC
ATAAATTGTTACAATGATACTGGTAGCATTGCAAAACGCTTTGGAGGTGGACC
AAAAAAACCGTAACGTCGAAAATGTTTTTGGTAAAGTTATGACGGAAGTTGA
AAGAATTTCACGTCACTGTGCCACTCAAAAGGATGGAGAGAAGTGAATTTG
AGCTTGAATCATTAAGGATTAAAGGAGTTGATGCGCTTGCACGAATGTGGCG
AATTTCCAAACATTGTGTTCTCTGATGAGAAAAATTTAGCCCAATTGGCAATT
CATGAACTCTGAAAACGGTCAAATTTACTTGACCGAACGCTCAGACGAGAAT
TTGAGCCTACATACGGCCACTCGTAGCAATTCCCCATAGAAAAAATAGCTTGG
GCCGCAGGGATCGCTGATGGGCGCTCTGCAATAGTTTACATCGAGCCTGGTG
TNNNNNNNNNNNNNNNNNNNNNNNNNNNNNNNNNNNNNNNNNNNNNNNN
NNNNNNNNNNNNNNNNNNNNNNNNNNNNNNNNNNNNNNNGTGGACACGT

AAACATTCCGGTCGTCGACCATGGACGTTCCAACAAGACTCAGCACCGTCTC
CTAAAGCTCATGTGAACCAAGAATAGTTAAAAAATCATGTTTCACATTCCACA
GACGCAAATGCGATGGACTATTCCATCTGGTCCATATTGTACAGCAAAGTGAG
GACTATAAATTATGCCAGTATGGATGCGCTGAAGAAATCGATTGTACGAGAAT
GGGCCAAAACACCACAAGATCACATTTGTGCAGCATGCAACTCGTGTATGGA
CCTTTAGCAGGCTATAGTTAATAGGCAAAGGTGGCCACATCGGGCTAAAGTG
AACGGATTCTGAAATTTTGATTATCCTCAAACAATTTTGTCTTTGAAATCACTA
AAAAATGATAACACACAAACAAGATATGGTGTTTTGAAAAGGTAAAGGCTTC
ATATCAGTAACCCTG

16 – Mariner16-Andl

Degenerated element

Short description

A total of 3 copies of this element were found in *An. darling* genome, the larger copies have an estimated size of 1471 bp. TIRs are 25bp long and TSD consisting TA nucleotides. The ORF finder found 6 short ORFs. However, there are long nondeterminate sequences (NNNN) which difficulties a more accurate analysis.

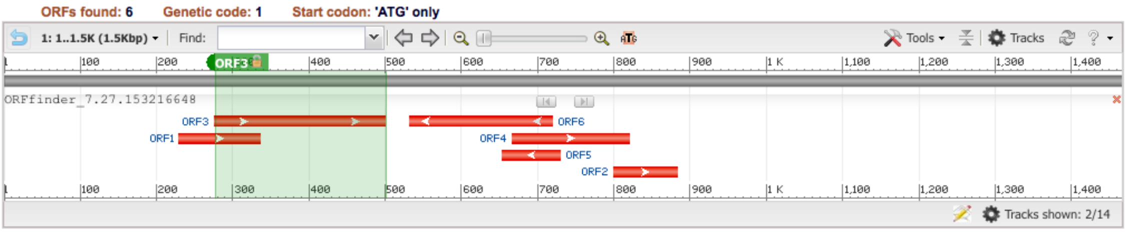

Figure 16 - Orf Finder scheme of ORFs detected in the Mariner16_Andl element

> Mariner16_ Andl (sequence) Red = TIRs

CAGTTGTGTTCAATAAATTAGCAGTGGGGTGGTTCAAAAGTTAAAAGTAATTT
AAAATGCTATTTCATACATTCGAATTCGAAATCTGTTCTGTGTGTATATAAAAA
GGAACAAATAGTAAACAAGAACATATGGTTTGCTTTTGAAAATCAACTCATTA
TTAAGTACAAATTTAAAATAAATAAAAATTATCTGTTCACAAATTCACAAAAA
TAGTAGTATTTCAACAATGCCGGTATTATCACCCGAGAATATTAAATTTGAAAA

GAAAAAAGATGCATTGTACTGGGATTGCTTTGTCCACTTCAATATTTTGCGGT
ATAGCCTTTATTCTTAATAACAGAGGCGCATCGCTTAGGCATAGAGCTCACAA
CTCAATGACATCGAGCGATAGGGATTGCAGCCCCAGTCTTCAAAACATTGCA
CCATTTTTCCTTGGGCCAGTCGACATGCTTTTTTGCAAACTTAAGACGGTTTG
TTACATGTGGCTTCGTCAATAATGGTACCTTGCGGGGTCTAAGTGCGTACAGA
TTAGCTTCCCTTAATCGGCGTCTGACTGTTACTGCACTCACAGACAACTCCAA
TACATCCTGCAATTCTATTGGAAGTCATTGCTCGTGGATCTCTACGCGATAAGT
TGGCTATCTTTCGGTCAGTTTTCTGGGATGTGCATCGTTTCCGGCCACAAAGT
TCTGCCTTTGCTTTCCATTTTAAGGTCATTACTAATCATTTCGAATCAGGTTGT
ACGATTTACCTTCCTTTTTCATTTTCTTATTAAATTCCGCAACTCTAGAGAACA
ATGTTTACCGCGCCTGCTATAAAAACAAAACCGACTTGTCTTTTCATGAATAT
TTTTGATTTATATATTCTTACCGATAGATGAACAATCAAAACTTGCCAATTTTTT
TTCAAAATATCCGCAAAAAACAATGAAATAGTATGGAATTGTTCTTCACTGCT
ACTATTGTGAACAGCGCAAAACAGACGNNNNNNNNNNNNNNNNNNNNNNN
NNNNNNNNNNNNNNNNNNNNNNNNNNNNNNNNNNNNNNNNNNNNNNNNN
NNNNNNNNNNNNNNNNNNNNNNNNNNNNNNNNNNNNNNNNNNNNNNNNN
NNNNNNNNNNNNNNNNNNNNNNNNNNNNNNNNNNNNNNNNNNNNNNNNN
NNNNNNNNNNNNNNNNNNNNNNNNNNNNNNNNNNNNNNNNNNNNNNNNN
NNNNNNNNNNNNNNNNNNNNNNNNNNNNNNNNNNNNNNNNNNNNNNNNN
NNNNNNNNNNNNNNNNNNNNNNNNNNNNNNNNNNNNNNNNNNNNNNNNN
NNNNNNNNNNNNNNNNNNNNNNNNNNNNNNNNNNNNNNNNNNNNNNNNN
NGGAGATCCCTTTACTACACACCAAAATGATCGATTGATAAAATAGACAACGC
ATGCTTGAATAATGAGCATTCAGTTACAGCGTGGTGCGCTACTGCTATTTTATT
GAACACAACTG

\

17 – Mariner17-Andl

Degenerated element

Short description

A total of 3 copies of this element were found in *An. darling* genome, the larger showing 886 bp. TIR are 29 pb long and TSD consisting TA nucleotides. The ORF finder found 8 short ORFs, indicating this element is not able to produce active enzymes.

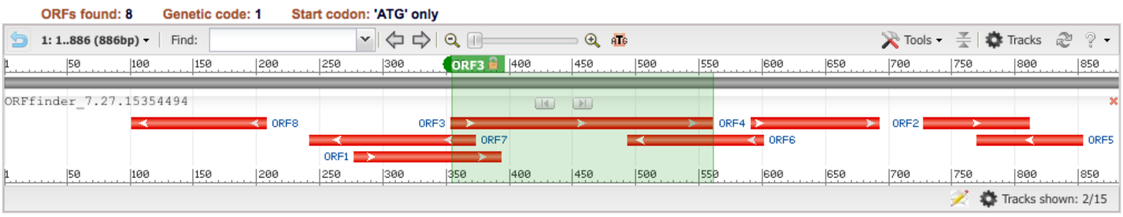

Figure 17 - Orf Finder scheme of ORFs detected in the Mariner17_Andl element

> Mariner17_ Andl (sequence) Red = TIRs

TTAGAGGTATGCGTTGAAATCCGCGGTTTTTTTAAATTCAATCATTTATATTTC
AAAACTCCTTTGCAACTGGTCATTGAAAGTATTGTCCATCCTTTTTCACTGCT
TTCTGCCATTTATCAGGCAATTTTCGGATCCCGAAGATCGAAAAAAATCAGCC
TCTATGGTGGCAGTGTAGTCAACTACCCATTTTTTGTTTACTTCCATAAAGAGT
GACCTGCCGTGCTGCCAAAAAATGCTTCAAGCAACCAAACAAGTATTATTGA
GAAAGAGCAATGTCTGGTGTATACAGTCGTGTGGGTAACATTTCGGCTGCGA
CGTTTTCTGAAAAGTTTTGACCGGTTTTGCAACATGCGGCCGAGCGTTATCA
TTAAGAAAAATTCGTTTATAATGTCGGATATCCCATTGCCATTTTCTAGCCAGA
GCTCGCACCAAATGGACCAGTTTCCGGAGGTTTTAATGCACGACACTCTTT
AAATCTTATTTTTTTCATTTTTTTGGTTGGCCCGAACGTTCTGTTTGTGTAAC
GCCGAAATTTCCGCTACTAATTCTTTGAAAATAGCTTTCACATTTTTGGTGTCG
TAACATGTTAACCATAAACTTAAACCAACAATCGATGAAGCTCAGCAGCCGT
TTTCGTCAAATGAAAAAAAGGGGCTTAAACTTTCCCGCAAAATTGACTTTTT
AAGCATCAAACTCGACAGTTTTAACTTGAGAAAAAATATGTTACAGTCAATAT
TTTGCACAGTTTGCACATATTTTGTTTTAAAGGCATTTAGTTATACTTTTAAAC
GATATAGCATATAAGGCTGTGAACCTTCCGCAAAAAAATAAAATGGCGTCTTC
CATTAGCAAACCGCGGATTTCAACGCATACACCTAA

18 – Mariner18-Andl

Degenerated element

Short description

A total of 42 copies of this element were found in *An. darling* genome, the larger showing 1268 bp. TIR are 215/225 pb long and TSD consisting TA nucleotides. The ORF finder found 5 short ORFs, indicating this element is not able to produce active enzymes.

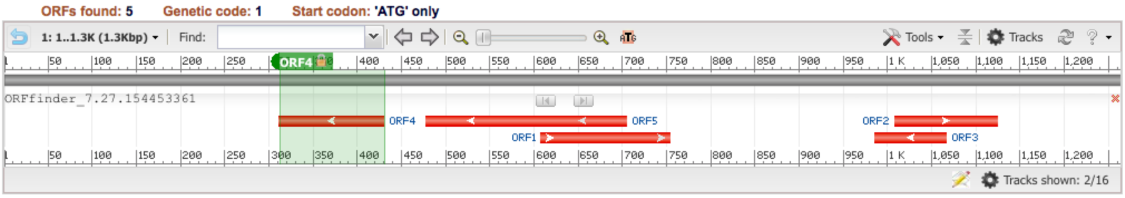

Figure 18 - Orf Finder scheme of ORFs detected in the Mariner18_Andl element

ORF 5

(Blast -Transposable element Tcb2 transposase-like Protein -Tribolium castaneum)

>lcl|ORF5

MDRIIKWYSTKDAFASGMEIKSKSSLDISSQTIQRRLGDGGLFSCKPAKK

PYINNEGITSFCDELFDLGCLNIKK

ORF 4

(Blast -Transposable element Tcb2 transposase-like Protein -Tribolium castaneum)

>lcl|ORF4

MKQARRPLYTRLNPQYCKKAIKYGRRCCWSEIPKERSTN

> Mariner18_ Andl (sequence) Red = TIRs

TACAGTGTCGGACATAAGTTAAGCAACTGGGTTAAGGTGTATGAAAAAGTGT

TTAAAATTTAGTTTTCAACCAAACAACCAACCAAAACCAAAAAGGGTACCT

ATTTACGATATTTTAAGACGATTTAAGTTATTAATTTTTCTTCGAATAGCACATT

AACAAAAAAGTTTCTTAAAAGGATTGAATGTTGATACGACATAAGTTAAGCA

ACTGAAAGATATCAATTCGTATTTGCTGATTGTTTCAGTATTTCGTTGCGAATC

AGTTATTTTTAATTAGAGCTACACTTCTTTTGGGCATAAATTCAGTCAATTTGT

TGATCGTTCCTTCGGAATTTCACTCCAACAACATCTTCTCCCATATTTAATAGC

CTTTTTGCAATATTGCGGATTTAATCTAGTATAAAGTGGACGTCTAGCTTGCTT

CATATCATCCGAATTAAACAGATCGAATATGGATTCATCACTAAAGAGCACTAT

TTTTTAATATTGAGACATCCCAGATCAAACAACTCATCGCAAAATGAAGTTAT

ACCTTCGTTGTTGATGTACGGCTTTTTCGCTGGTTTGCAACTGAATAACCCAC

CGTCTCCCAATCGGCGCTGAATGGTCTGGGAACTGATATCCAAGCTTGATTTA

CTTTTTATTTCCATTCCTGATGCAAACGCATCTTTGGTTGAATACCATTTGATA

ATGCGGTCCATTTTGGATGAAGTTTTTCAAAGACGCCTTTTGCCTTATACGGG

TTATTAAATGAGAAACTACTGATTTATCTATGCCATAAGCTTCACTAGTGTATTT
CTAAGAGCTTCCCAGAATCGCTAGTTTCATTTCTATTTTGTTTTCAATCGCTAG
AATAGATATTACGCGACACTGTTACGTTTTGAATGTAACCGAGTGTGTTGGGC
GTTCAAAGGCAAAAAGAATACTAAAACATCATAAACCTTGACAGATTTAGCA
AGTTTACACACCAATAGGCTACTACTATGCAGAAATTCCGCATGTGATGATCA
ATATTTTCTTATTTTTGCTCCAAAATAGCCAGTTGCTTAACTTATGTCGCATTAA
AAATCTATGCCTTTTTGAAACTATCGTGTTAATGCGCTATTTTAAGAAAAATTA
ATAACTGAAATCGTCCTAAAATATCGTAAATAGGTACTCTTTTCAGTAGCTGA
GTTGGAAATTGTACAGTTTGGTTGAAAACTAAAATTTTAAACACCTTTTCATA
CACCTTGACTCAGTTGCTTAACTTATGTCCGACACTGTA

19 – Mariner19-Andl

MITE

Short description

A total of 9 copies of this element were found in *An. darling* genome, the larger showing 792 bp. TIR are 17 pb long and TSD consisting TA nucleotides. The ORF finder found 5 short ORFs, indicating this element is not able to produce active enzymes. The short length, conserved TIR and TSD suggest this element can be a MITE.

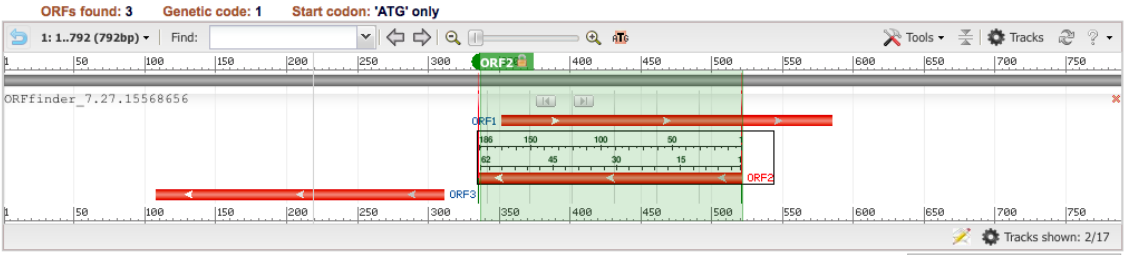

Figure 19 - Orf Finder scheme of ORFs detected in the Mariner19_Andl element

ORF 3

(Blats - Mariner Mos1 transposase - Acromyrmex echinatior)

>lcl|ORF3
MKYYPTRCIHQTMFYLNTACLVTYIFFAYRTSLIYARIEKWLLDKITLKN
EYFFRHRIRKLTERWKK

> Mariner19_ Andl (sequence) Red = TIRs

TTAGGTCTCTGAATTAGATTTTTCGAGTTTTTGGCTCAACAAAACCCATCTATA
CTCAAAACCAATAGAATAATTACATTAATCCAAATATTGCCCATCGTTGGCCTC
TACTTTTTCCATCTTTCAGTTAATTTTCGAATTCTATGTCGAAAGAAATACTCA
TTTTTTAAAGTAATCTTGTCAAGTAGCCATTTTTCGATTCTTGCATAAATGAGT
GAGGTTCTGTACGCGAAAAAAATGTAGGTAACCAAACAAGCTGTATTCAGAT
AAAACATTGTCTGGTGTATACAGCGAGTGGGGTAATATTTCATTTGTGACGTT
TTCTGAGAAGTGGTTCACTGATTTTGCAACATGATGTCGTGCGTTATCGTATTT
AAAAATAAGTCTATCATGTCGGTTATCCTATTCTGGCCGTTTTTGGTTGGCCGT
TTTTGGTTCTGTGTCTGTAACGTAAAAATCTCCGGTTCTAATGATTTGAAGCC
AGTTTTCACGTTTTTTGTGTGTCGTAACATGTTCATCATATGCCTAAACCGACA
AACTATAACTTTTAGCGGCTGGTTTATTGAAATAAAAGAAGGTTTTTAAACTT
CTCCGCAAAATTGACTTTTGAGACACAAATCTCTACATTTTTAACTCGAGAAA
AATATGTTATAGTTAATATTATAAGCAGTCACTTATTTTGTTGTTGTAAAGGTGT
TTAGTTATTCTTTCAAATGGTATAACATAGGGGGCTGTGCAACTGACCAAAAA
TCAGTGGCGCCAGCTTTTAGCAGAACTAATTCAGGGACCTAA

20 – Mariner20-Andl

MITE

Short description

A total of 49 copies of this element were found in *An. darling* genome, the larger showing 759 bp. TIR are 25 pb long and TSD consisting TA nucleotides. The ORF finder found 6 short ORFs, indicating this element is not able to produce active enzymes. The short length, conserved TIR and TSD suggest this element can be a MITE.

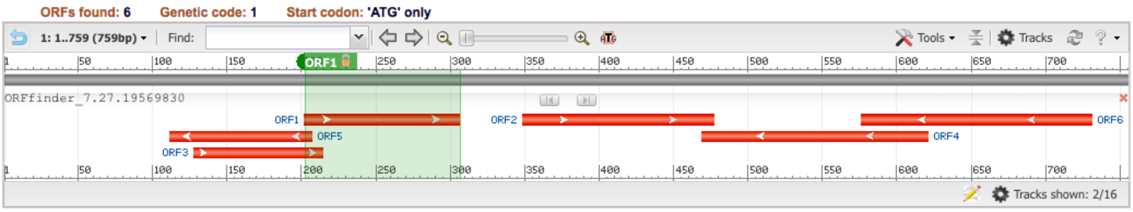

Figure 20 - Orf Finder scheme of ORFs detected in the Mariner20_Andl element

> Mariner20_ Andl (sequence) Red = TIRs

AAGCCCAGTCCATTTAGAACCTGGAATTGGAGCTGTCGCAACAATTGGAGAT
CCCATACCAAATCATTATATTACGGGCAAATTTATCTCCGTAAATAAACCAAAC
CCTTCCTAAATCATTCCATCTATGAACGGCAAAGTCAAATCTGTTGCCTGGTAT
TTTTTAAATTCATTTTTACGTGTGTTCCACCGTCCATCAAAATGCATGGTTTGA
ATTCGGTCAATAGTTGTTCATACAGATTCCCTGCACGGATTTGGGCAATCAAG
CTGTGCTCTAGGGTCCTATTTGAGTATTTTCTGGTATGATACGACCTTAAAATT
TCCTTTAAACATATTTGCCGAACTGTTATGACGTCTGCGGTGAGTTTTGTGGT
GGAATCACGCTGGGAGATCCCAGGATTGTTGATTTACTTTTGTCTGCAGAATT
GGTTATTTGTTACACTTTTCAGCTATTTTATTTTGCTAGAATCAAGGTAAAAGT
CCTCCCGTAACGTTTGCATACGGTATTTTTAGTTGAGTTTGGAAATTGCAAAA
CTTTCGCGATTTTTCTCCAGCCAAATTTTTAATTTTAAGTTTTAATAATAAGTGT
GCAGATTTATTTGCATCGAGTAAGTGCCCCATTGACAGACCCGTCGCCAGAA
CAATTTCGGGTCTATTAAACTATAATAGACCTGGTTGCATACATTTTGCATACG
GGTAACAACTAGAGGCACTGCAGCGATTAAAAACATGTGTCCAGATTCTAAA
TGGATGGGGCTT

21 – Mariner21-Andl

MITE

Short description

A total of 1 copies of this element were found in *An. darling* genome, the larger showing 796 bp. TIR are 30 pb long and TSD consisting TA nucleotides. The ORF finder found 6 short ORFs, indicating this element is not able to produce active enzymes. The short length, conserved TIR and TSD suggest this element can be a MITE.

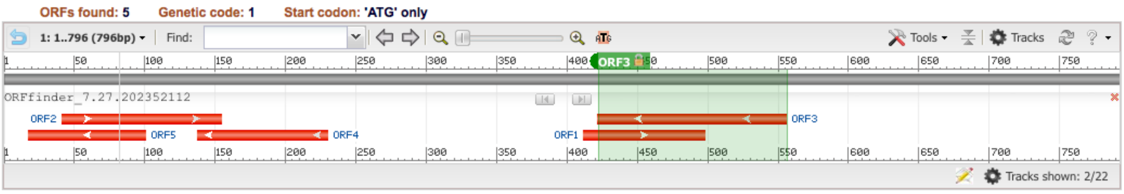

Figure 21 - Orf Finder scheme of ORFs detected in the Mariner21_Andl element

> Mariner21_ Andl (sequence) Red = TIRs

TTAGATTGTTTCATGTGTTATTGCGTTTTTTCTATATACAGATGGAAATGACAAT
TGTAATAGCGATTTCTAGCAAAGTTACAAATGTATCCTTTTGCCATTTATCAGT
TGTCACTTTTAGCTTGTTTTTGTTAACGTTAGCAAGAATTTGGTAGGAAAAGA
GTGCGCGTAATTTCGTTTATAATTGTGTGTGTAGCGTCACTTTCAATGTGGAGT
ACTAAAGCAAACATATTCGACACATTTTAATGTATTACTTTTAGAGAAGCAAA
TCTGCCGGGCATGTTCCAAAAATAATATGTGATAAGTATGGTAGGGAATCAAT
AAAATAGAGTTTTTGTCAAAATTGGTTTAAATACACATGTTGCAAAAAAAAG
AAAGATTAAACAAATGAACAATAATGGAAATAATTGAATGTCTACTGTTAAAA
ACCTGGAACTGGAATGGAAGCTACTAGAATGCAACCCTCCACGAGCAACGT
CCAGAAATTATAAATCATAAAAAATTTCTGCCAGGACATTGCTACAACGTATA
CATTTTTAGTGCCCTCGCCAAACATTTAGAGAACTAAGCTGGGAACTTTTGAA
GTACCCGTCATATAACCCTAACCTTCCACCATTAGCGTTAGTAGCGATTTTTGC
AGATCTCCCAGTGTGGTAAAGCTCTGATGGAGAAGATGACACTGTTAAATCG
AACTTCTCTCAGTTTTTAGACGATAAAAACGATAGATACAAAAAGAATTTAAA
AAACGGTATTTACTTTCGAGGACAAAAAAGAAATTACTTATGAAAGAACCTA
A

22 – Mariner22-Andl

Degenerated element

Short description

A total of 2 copies of this element were found in *An. darling* genome, the larger showing 1755 bp. TIR are 25 pb long and TSD consisting TA nucleotides. The ORF finder found 15 short ORFs, indicating this element is not able to produce active enzymes.

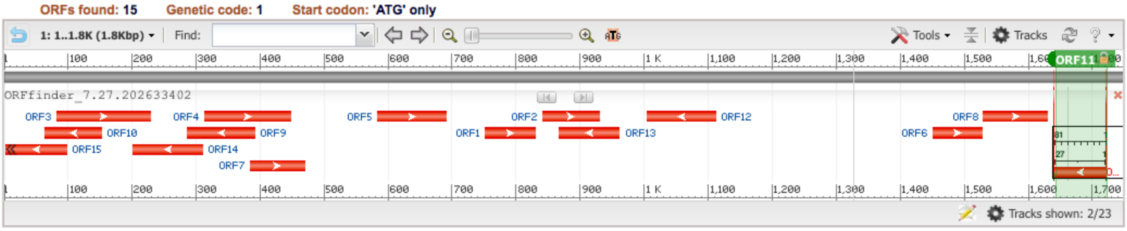

Figure 22 - Orf Finder scheme of ORFs detected in the Mariner22_Andl element

> Mariner22_ Andl (sequence) Red = TIRs

TTAGGTTGTTTCATAAGTAGTTGGGGTTTTGTAATCGATAGTAAATACCGGTA
TTAAAATACTCAAAACGAAGACTGACCAATGATATATATTTCATTTTGTTCTCT
GATNNNNNNNNNNNNNNNNNNNNNATCTTCTGGATTTGCCAGCCATGGATCTT
CCCGATTTTATCTTCAAAAAAACTGAACTGAAGTCCTATTTCATATTGCCATCC
TTCTTAAAAGTTTAAACATACGAAGGGTGAAACGAAATGGCTGCCTGAGGAA
TGTTCCCAGCCAAGTTCTTTCAATTTTTGCCGATGGCATAAACATGTATGCGG
TCTAGCGATGTCCCGGTGCAATGTGAAATCTTTGCAATTGACAAATTCTGGTT
GTTTCTCGTGAATGATTGCATTCAATTTGACATATTTGTTTCAGTTTGTTTCATT
CTTCAAATCCCACCTAACTAAGAGCATGTTTCTCCCGTGGTGAATATACCCTT
TGGAGGTCGTTTGAAGTGACTCATCACGCTCCGAAAGCGATCATTTTCTATTG
TTATTATTGTAAACAGTACAACAGTGCAACAATGTAAACTTTCAATATCTATGA
TGATTAGCTCGAAAATTGATCGATTTCATAACGTCAAAAAGCTAATTACAAGT
GTTAAAGTCGTTTGTAAAATGAATTTCATTCAATTGATTATACCATCCTAATATC
AAGCTGTTTAATGACCAAAACCTATTGAATATGAGGTCTTTCTTTTATCGTTAA
TATGTCTGACGATTTGTCAGATATAACCTTTCTTCGACCGAATGTACTGTTATA
TGACGATGTGTCTCGGTTAATGCTTTAATTTCGTCAAATGCTTTCGGACGGTT
TGAAAGTAGTTCAACTTTTAGGGNNNNNNNNNNNNNNNNNNNNGTCCCGGT
GCAATGTGCAATGTGAATTTTGAATTTGGTCGTTTGGTTCCTTGAAGCGGCTC
ATAAGTTACCACAAATGTCCTTCAACACGCACCTAGCTAAGAGCTTAATTTTC
CTTTGGTGAATCTACCCTTTGCAGGTCGTTTGGACGGATCACGCTCCGACAGT
GATAATTTTCTATTGTTTTTATTAAAATCAATGCCACTTTGAACATCTCTGATGA
TTAGCTCGAAAATTCGCTCGATTTCATAACGTCAAAAATCTAATCACAAGTGT
TAAAGTCATTTGTAAAATAAATTTCATTCAATTGATTATGCCATCCTAATATCAA
GCTGTTTAATGACCAAAACCCATTGAATATGAGGTCTTTCTTTTATTGTTAATA
TGTATAACCTTTCGTCGACCGAATGCACTATTATATGACGATGTGTCTCGATTA
ATGCTTTAATTTCGTCAAATGCTTTCGGACGGTTTGAAAGTAGTTCATCTTTG

AGTGTTTCTTTCTTCCAGATCGGAATATTACTAACCAATTATGACAAAAACTG
CCTTTTAATGATTCCGTACCTCACTCATCACGTATTTTGCGCAACATGCGCTG
CGGTTTCTCTTCTCTAAAAATTATAAAGTAATATGTGCCGAAATTTTCGCTTTG
GAATCCGTATTAAAAGTGACGCCACACTTACAAATATCAACGATATTATGCCC
ACTTTTTTCCCAAGAAAACCTAACTAAAAGTGACAACTAATGAATTCTAAAA
GGATCATTTTTTGCATTGCTAAAAACCGTTAAACAATTGGTCATTGGATTGGA
AAAATTGGTCATGAAAATGGACGCATCTACATATGAAACAACCCAA

23 – Mariner23-Andl

Degenerated element

Short description

A total of 200 copies of this element were found in *An. darling* genome, the larger showing 1212 bp. TIR are not found. The ORF finder found 9 short ORFs, indicating this element is not able to produce active enzymes.

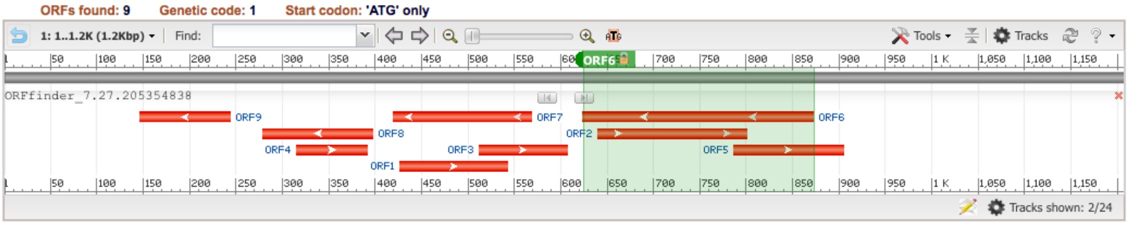

Figure 23 - Orf Finder scheme of ORFs detected in the Mariner23_Andl element

> Mariner23_ Andl (sequence) Red = TIRs

AAGCATGGAACAGTTAATATTTGGCCGCACAAAATAGGTCATATCTCAGCCAA
AAAATGACGTATAAAGAAAAGAAAGGTGTCATTTTGTAGGTTTTCTTATTGCC
TTTAATAAGAAATATTGATGCAAATTATTCATTAGTTTTTTATATGACTTTTTCA
ACTTTTACTTTGACATCACCCAAAAATNTTTGCACAGTACTGTAGGTCACATC
ATTTGCCCTCTTGTTGCACATCACCCTCATTTCAGTTGGTTTTTATACATTTCT
GATATACTTTTTAAGTTTCCTTATGATAATTGTCCAATAGAAATCGATGGAACG
AAAATCCAGACATTTTGNTGGATTGATAGTTTTCCCTATGAAATCGATCTATTT
GAATACAAAAAATTAACGCCATACCTTCGGCAAGAACAGCTACCTAAATCAT
GCCAAACTTACACAGGATCATTGGTGGACAAATGNAGGACAAAATCCTTTTC
TGTAGACACTCTTTTCTTGCACAAATTTCGAATGAACGTTGCCCCAATGGTGA

AAACATTTGATTTGTCGCNACAATNTCANATCCCATGCCAAATCATCATCTTA
NGGGCAAATTTATCTCCGTAAATAAACCAAANCCTTCCTAAANCATTCCCTCT
ATGATTGGCAAAGTNANTNNTNNNNNNAANNNTTGTNNNTNCAGNNTNNNN
GNANNNNCAATCAAGCTGTGCTCNAGGNNNCTNTTTGGNTATTTTCTNGCNT
NTANGACCTTAAAANTTCTTTTAAACATATTTNCCNAACTGTTATGNCGNCTG
TCGTGAGTTTTTNTTNGTNGAATCACGCTGAGAGATCCCAGGATTNTNGANT
CANTTTTGTCTGCAGAATCGGGCATTNGTTACACTTTTCGGCTGTTTTATTTTN
CTAGAATCAAGGTAAAAGTCCCCCAGTAACGTTTACATATGGTATTTTGAGTT
GAGTTTGNAAATTTCAAGACTTTCGNGGTTTTTTCTCCAGCCAAATCGANTTT
TAATAGTGAGTGTGCAGATTTTTTTTCNCCCTTCGCGTTCCATCGGCGATAAC
TTTTGAAGGCGTGGATCAATTTTGACAAAAAATTTACCACTAAATAAACAGA
CTACAATGATTAAAACGCTGTCAACGGAATGGCAAAGTGACCACTAGGCGCG
CTGCAATGATTAAAAANAAGCGGCCAAATATTAACTGTTCCANGCTT

24 – Mariner24-Andl

Degenerated element

Short description

More than 100 copies of this element were found in *An. darling* genome, the larger showing 394 bp. TIR are not found. The ORF finder found 3 short ORFs, indicating this element is a fragment of a mariner element.

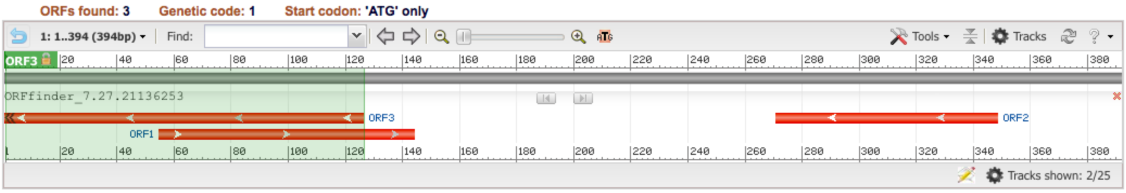

Figure 24 - Orf Finder scheme of ORFs detected in the Mariner24_Andl element

> Mariner24_ Andl (sequence)

GCCGTGGCCACACGGGGCGAAAATTTGCGCGCAAATTTGCACATTAATGCCA
AAATGTTTTCGCTTCGTGTGGCAGGGGTAAAAACCCGAAAATTTATGCCGAA
ATGTCAAACGTATTGTTTTCATCTGTGTTTTGGCCGCTGAAGTTGATTTCCGA
ATAAATTTTGCAGTTTTTAACGATTTTGTTGCTGTTGCTGTTATATTTATATTAA
AAATGCAAAAACAATACGAAAAGAACCTACATTTTCGTAAATAAAGCGTTCG

ATAGCGTCAAGGGTTCAGCGAAAAAGTCCGCGGACGTATAAAAGTTTGACA
GCGTGTAAGGGTTAAGCGAAACCGTTTTGGCATTAATTTTTTCGTTTGCGCTA
GAGTTTTCGCCCCGTGTGGCCACGGC

25 – Mariner25-Andl

Degenerated element

Short description

More than 100 copies of this element were found in *An. darling* genome, the larger showing 394 bp. TIR are not found. The ORF finder found 3 short ORFs, indicating this element is a fragment of a mariner element.

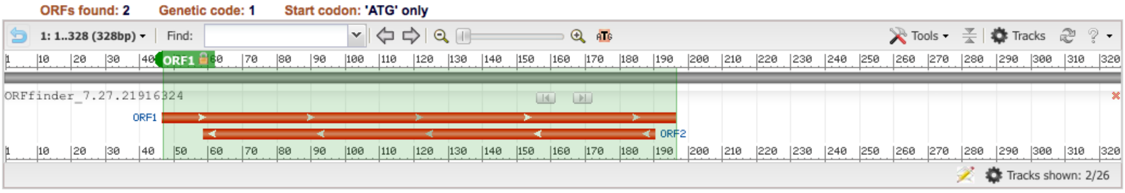

Figure 25 - Orf Finder scheme of ORFs detected in the Mariner25_Andl element

> Mariner25_ Andl (sequence)
GCCGTGGCCACACGGGGCGAAAACTCTAGCGCAAACGAAAAAATTAATGCC
AAAACGTTTACGCTTGCCGTTTACATGCTGTCAAACGATTATAGGTCCGTGGA
GCGTAAAACCTAGCGTAAAAGCTGTTTGCAAACGGAGGGCTCGCAATATGTT
CTTTTTGTGGTTTACATCGATAGTGAGTGATCATTGCTAGTGTGTTCAATCCTT
TTCTTCCACAAAACGATTTTAATTTCCTCAACCCGGCCACGTAAACATATCGA
AGCGTAAACGTTTTGGCATTAATTTGTAAATTTGCGCGCAAATTTTCGCTCCG
TGTGGCCACGGC

26 – Mariner26-Andl

MITE

Short description

A total of 21 copies of this element were found in *An. darling* genome, the larger showing 702 bp. TIR are 28 pb long and TSD consisting TA nucleotides. The ORF finder found 3

short ORFs, indicating this element is not able to produce active enzymes. The short length, conserved TIR and TSD suggest this element can be a MITE.

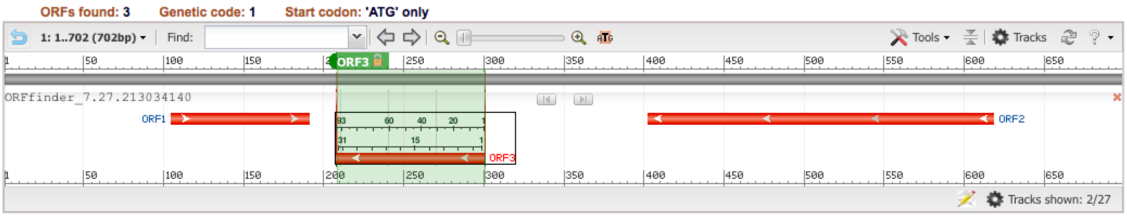

Figure 26- Orf Finder scheme of ORFs detected in the Mariner26_Andl element

> Mariner26_ Andl (sequence) Red = TIRs

CACTCTGTCCAGTTACTATAAGACCACCTGTGATTTTAATAGTTTCTCACTACT
GATAACAGTAACAATTTCAATTCGTATCATTATTTGTTGGTATTGGTGAGATGT
ACTTACAGTGCAATTATCGCATTTTTACGTGCATATACTACACAAATACACGCA
TTTCTTTTGTTGTCGATCCGTCTAGTTAGTATAAGAACAGTTCGCTTAGTCCGC
GTTTTTACATTTTTGGAGGTAAATTTCACAAAATGCCCAAAGGAAAAGTATTA
ACTGACCATGAGAAAGGTCAAATAAAAGCATTTTGACGAGAGAATGTAGGA
ATACGAGAGATTGCACGACGGTTAGGCCGATCTCATCAGGGGGCACTAAATT
TCCTAAAAAAATTCCGCAAGCTACGGAACATCATAAGGAAACACGAAAAAC
AAAGCTGTCTAACCGAGACAAAAGACAAATAGTGAGAATAGCTTCAAATTCT
TCGATCTCGTTAGCTAAAATTATTTTTTTAAATTCGCTTAAGTTAGAAGTGTCG
AGAGAAACAATTCGCCAAGTTTTAGTGAATAGCCCGTTCATAAAAAGAGCTA
AGAAGACAAAAGCCCCTAATCTCACACCGATCCACATCGAAAAACGATTGAC
CTTTGCCAAAAATAATATGAGCCGTCAATGGAATTCGGTACGGTGGTCTTATA
GTAACTGGACAGAGTG

27 DNAUnknown-Andl1

Degenerated element

Short description

More than 100 copies of this element were found in *An. darling* genome, the larger showing 555 bp. TIR are not found. The ORF finder found 3 short ORFs, indicating this element is a fragment an unknown element.

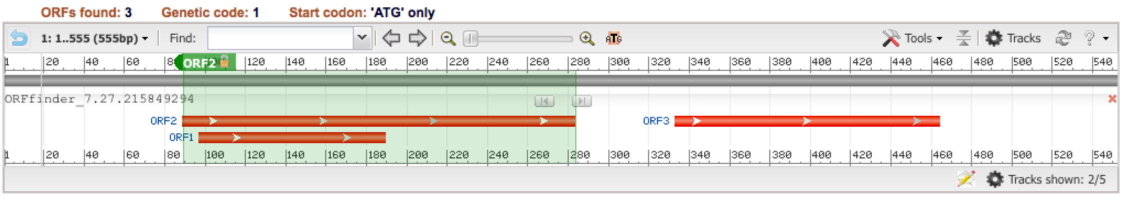

Figure 27 - Orf Finder scheme of ORFs detected in the DNAUnknown-Andl1 element

> DNAUnknown-Andl1 (sequence)

CAAGGTACTTTAAAAAGACAGGTAGCTTTATGCAGAACAAATCCCCGATCGT
ATTTTAGAAAAAACTTCAGAGTTTGACATTCACGGGATGGTGGGATGTTTACT
TCGGGAACGCTCTTTTCGGAGCTGTTTTCGCTTTTCCGTGGTATTACGACAGT
TAAAATTCACGAAAAGTGGCACGAAAGTTGAAGTTTATGCAAATATTCCCAA
AATTCTTCCTAGTGTTTCAATATGGTATCGGTCCGTAGGTCTTGCCACCCGGCC
TAGAACAAGAGCTAGCTAGCGTTCCTGGACCGTGCTCACTCGCTGTAAAACA
CAACTCGGTCGCTCGAATGATCTTAAATAGAGAGATGAATCATTTAAACATTC
GAAAAAGAGTGAAATCAGGTACTTTCCCTGATAAAACCACCAAACTTGCCCA
TATCTTCTTCTTCTTCTTCTTGAAACTCACGTGGTTTTGTTGATAAAAAGCGCC
CTGCTACGAATGTCAAACAAATTCAAAATGGTGACCTGTCAAAGCGGTTAAG
AAACTACCTGTCTTTTTAAAANACCTTG

28 DNAUnknown-Andl2

Degenerated element

Short description

More than 100 copies of this element were found in *An. darling* genome, the larger showing 234 bp. TIR are not found. None ORF was found by Orf Finder, indicating this element is a fragment an unknown element.

> DNAUnknown-Andl2 (sequence)

GGTAAAAGACTGAAATAGGCCCCCCACTCACGGTGAGTTACTGAGTGACCG
AAAAAATCGTTAATTACCACTTTTAGATAAGCAAACCTTCATAAAAATATGC
GGAAACGTAAATAAATGTATTAGGGGAGGGGGAGGGGTGGAAAAGTTACA
TTTTACCAGTAAAACAACGATTTTTCGGTCACTCAGTAACTCACCGTGAGTG
GGGGGGCCTATTTCAGTCTTTTACC

29 DNAUnknown-Andl3

Degenerated element

Short description

More than 60 copies of this element were found in *An. darling* genome, the larger showing 540 bp. TIR are not found. The ORF finder found 5 short ORFs, indicating this element is a fragment an unknown element.

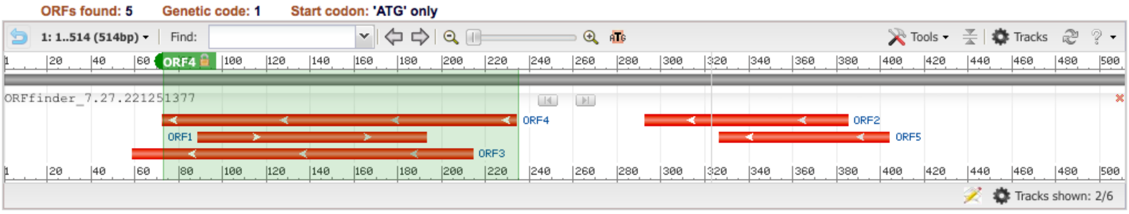

Figure 28 - Orf Finder scheme of ORFs detected in the DNAUnknown-Andl3 element

> DNAUnknown-Andl3 (sequence)

GGTAAAAAACCAAAACATACACCTCAACCGATTCAAATAATTTAGCGGTTAT
CCATTTCAAAATTTTGCCGTTAGATAACCGATAGAATGAGCCGGACACAGAA
ATACATGTGGAAAGTGGAGGAGGGGTGGGGAGGGAGCGAGTACATACCTAG
TACATCGCTTGTACCTCCCCCCGTCCCTTCCAGATAGATAAAAAGATGATTTA
GACATAATAATTGTCCCAAAAACATACACACCTTTTCAGTTAAATATCTTTGAA
AAATACATTATAAACGTTAAATAGTGCTATTACGGAAGAGGGGAGGGAGATG
CCATTACATTTTCACTGTCGAAAGATAATATTTGGGTAGTTTTTCGCACCATAA
ATTGATTTAATCTCATGTTTAAGGTTCTTGTTCATTCCACTGGTTATTTAACGG
CCAAATTTTGAAATGTTCGGCCCCTAAATGTATGCAATTAGAATCGGTTAGCC
TACCGAATCGGTTGAGGTGTATATATTGGTTTTTTACC

30 DNAUnknown-Andl4

Degenerated element

Short description

More than 40 copies of this element were found in *An. darling* genome, the larger showing 478 bp. TIR are not found, TSD shows TTAA nucleotides. None ORF finder was found by Orf finder software, indicating this sequence is a fragment an unknown element.

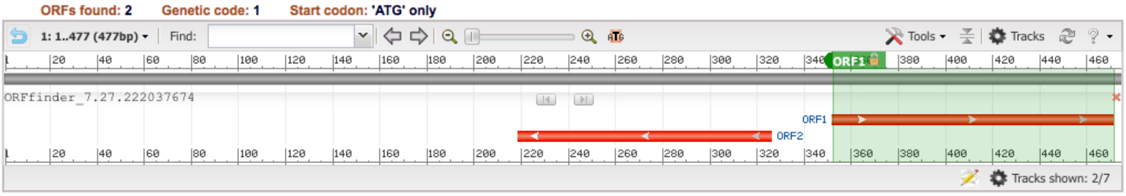

Figure 29 - Orf Finder scheme of ORFs detected in the DNAUnknown-Andl4 element

> DNAUnknown-Andl4 (sequence)

GCTGCTTTCACATCGAGCGCGTTTAAACGCCACTAATAGACGCAGCAGGTTT
TTTGACGCAGAAAACACGGTTGTATCACATCAAAATATATGGAAGTGTTCACG
TAACGCACCGAAAGCTATTTTTTGGAGCTTTTGAAGAGCTTAAAAGTTGGCG
TTTTTTTTCGGATCTGTAAAAATCTACAGATTGTGGCAGGTTCTGAGCCGACT
GTAGCAGGCTACGCAAGCAAGCCAGCGTTGCCCTTTGGTTTTGCAATTTGTT
TTGGCAGGAATGTGTTGAAATCCGGGTAATAACTGTTTTGCGTGAAGATTAAA
CTATTTATCATGATTTGATAAGGTGGCGGTAGTGATATGGTGCTGTTTTTCGTG
TATTTTAAATCGGCCTTCGCGCAACCTAGGTGTGAACAACAAATTAAGCTTTT
GCGTCAAAAAACGCCAACTTTGGCGTTTTTAAAAACGCGCTCGATGTGAAAG
CAGC

31 piggyBac1_Andl

Truncated element

Short description

A total of 27 copies of this element were found in *An. darling* genome, the larger showing 2954 bp. TIR are 19 pb long and TSD consisting TTAA nucleotides. The ORF finder found 20 ORFs, 3 larger ORFs codify 226, 127, 92 aa of piggyBac transposase, showing these copies are truncated.

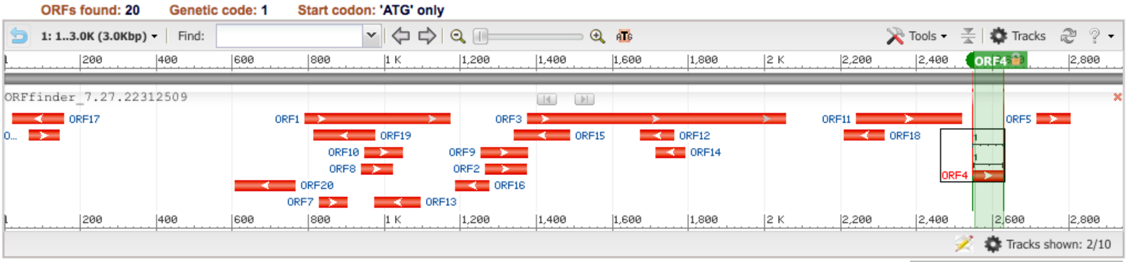

Figure 30 - Orf Finder scheme of ORFs detected in the piggyBac1_Andl element

(Blast - piggyBac transposable element-derived protein 3-like -Trachymyrmex septentrionalis)

>lcl|ORF3

MKEDKVAPIRDIWTMLNNNLAKYYKPSEYLTIDEQLFPYRGRTKIKQYIL
SKPAKYGIKVWWICDAVNSYPLNGQIYTGKSSTGREKGVGERVIKDLAAA
YRGSGRNITADNFFMSMPLAEFLLSWNLTIVGTLRKNKPYIPPAMTVLKN
REPFSSVFAFKDKITMCSYMPKKGKSVIMLSTMHQEAKIDDGIKKKPEII
EFYNWSKAGVDTMDKMLGRYSTQRCT

>lcl|ORF1

MSCVYIFKLFQEHEYGYDSEDEDETDVESDQERNDNEVNLGEDGDMTAKD
GTKWTKTPAVEHQAALRNIVRQKSGPVKNTEMLSIFELFKLYFSPEMADI
IIRHTNKKATRCYDAYNLRNPTKKPLK

>lcl|ORF11

MRNFSTKLAIESILGKAIHTNAVPSSDSELSQCHLAAKVIDSTGRKKITG
VCYMCLGGQSKHRRKTRKSCSICYLPICNEHCIDQTICNQCK

> piggyBac1_Andl (sequence)

CCCTCGGTTGGGCACCCGGCGTTTACCCCTTCGTTGGGCACCCGGGTACCCG
GGTACCCAACTCATGTGCATAGCACAGTTGATTGATCGTTTCGCGGCATTTAT
CGATTTTATGATCAAAACTAGTTCGCCACAACATAGTTAAAGGTCTTGGCATC
TAATTTCTGTTTCACAATGACAACAATACCAAACAAAAACAATAACAAAGCA
AGATAATTGTATAATTAGCCGAGCGCAAGGTCATTGCCAAGTAGATCGTAAAA
ATCGCAAAGCGAAAGAAGGGGTTATTTCTATTTATTATTTTGTCGGATAAAATT

GGCCTAGTCAAAACATCAATAATATTATTTTAGTCTAAACAAATCAATTCTTTG
TTGTGTTTTACTTGACTTTCTTTGGTTGTTCGCTCGATATCAAAGAGTCGAGTA
AGAGTCAGTCGTACAAATTCGTCTGACAGAGTAGTCGCTTTTTGGATTTTTTA
TCTGCGTGCTAGTAACTTGTGTTCGTTGTGGTAAAATATGGATCTTCCTATATA
TAAAAGGTGTGGTGTACTTTCATTATATGCAATTTAAACAATACTGTTGTGCAA
TGGTATTTGTTTTATTAGAATTACCCGCTCGCAGAAGGTGAAATTTGAAAATT
CGGTCGAACCAGAGGAGTGGGAAAATGACGATGACGATGATGATGATGATG
ACTACATTCCTCTAACTGAAATACCATCAGATTGCGACTCTTCTGGTAATTTTC
TTTTTTAGTTTTCGTGAACATTATCAATCGTAATTCACGAACATAATGTCTTGT
GTTTACATTTTCAAACTATTTCAAGAACATGAGTACGGTTACGATAGCGAAGA
TGAAGATGAAACTGATGTCGAAAGTGACCAGGAAAGAAATGACAATGAAGT
AAATCTGGGTGAAGATGGAGATATGACTGCAAAAGATGGCACTAAATGGACG
AAAACACCAGCAGTGGAACATCAAGCTGCTCTCCGCAATATTGTGCGCCAAA
AAAGTGGCCCCGTAAAGAATACGGAGATGTTGTCCATTTTTGAATTATTCAAA
TTATATTTCAGTCCAGAAATGGCTGATATTATCATACGCCACACGAACAAAAA
GGCAACACGATGTTATGATGCTTATAATCTACGGAACCCAACAAAAAAACCA
CTGAAGTAGAAAAATTTGGAAATTAATGAATTGTATGCGTTCCTCGGTGTGTT
AGTAACGTCTGGTGCCAACAACTCAAATACCGACAATGTGCATGATATGTGG
CAGTCATATTCCTACCCCCTGTACCGTGCGGCGATGGGCAGCAAACGCTTTCG
TTACATTAGTCGATTTATTAGATTTGATGATGCCAATACTCGGGAAAAGTGAAT
GAAGGAAGATAAAGTTGCTCCAATACGAGATATTTGGACAATGCTAAACAAC
AATTTGGCAAAGTACTACAAACCGTCAGAGTACTTAACCATTGACGAACAGC
TGTTTCCATACCGAGGAAGAACTAAGATCAAGCAATATATTCTATCAAAGCCA
GCAAAGTACGGAATAAAGGTTTGGTGGATTTGTGACGCAGTAAACTCTTATC
CACTAAATGGCCAAATTTACACTGGGAAATCAAGTACAGGCCGAGAGAAAG
GCGTTGGAGAAAGAGTGATTAAGGACTTGGCGGCTGCTTACAGGGGATCTG
GCAGAAACATAACCGCTGATAATTTCTTTATGTCAATGCCGTTAGCAGAGTTT
TTGCTCTCCTGGAACCTAACCATTGTTGGCACATTGCGGAAAAATAAGCCATA
TATTCCACCAGCTATGACTGTACTAAAAAATCGAGAACCATTCTCTAGCGTGT
TTGCTTTCAAGGATAAAATCACAATGTGCAGTTACATGCCTAAAAAGGGTAA
ATCGGTTATTATGCTTTCAACAATGCATCAGGAAGCCAAGATCGATGATGGCA
TAAAGAAAAAACCAGAAATAATAGAATTTTATAATTGGTCAAAAGCTGGTGT
GGACACTATGGATAAAATGCTCGGCAGATATTCTACGCAGCGATGTACTTAAA
GATGGCCTTTAGCTTTTTTTTTAACTATGTTTAGATTATCGTCTGTGTTGCCTCA

TACATAGTATACTGCGAAAACAATAAGAAAGCTGTGGTTAAAACGCACGAAC
GACGCTTAATTTATCGACAATTGGGCAGAGAACTATGTAGTTCATTTATTGAG
ACTCGATCGAAAAATCCTCAAATAATGAGGAACTTTTCAACAAAACTAGCGA
TTGAAAGCATTCTAGGTAAAGCAATACATACGAATGCTGTTCCATCATCTGAT
TCGGAATTATCTCAGTGTCATCTAGCTGCAAAAGTGATTGATTCAACCGGGCG
AAAAAAAATAACCGGCGTTTGTTATATGTGTCTTGGTGGTCAATCGAAACACC
GCCGAAAGACACGTAAGTCTTGTTCAATTTGTTATTTGCCAATATGCAACGAA
CATTGCATTGATCAAACGATTTGCAATCAGTGTAAATAAACAAGTTCAGAAA
ATATTTAATGTTTCAATGGCATATATAATGATTCATCTCCGTTCCTTATGTAATAA
AACAACAACAATAAACAATTTAAAAGTAAAAAAATGTTAAATTCTACTTAATT
ACTCATTAATTGGTCAGCGCACATCAATAAATGTTGACAAAAAGTGTTATTTT
GTGTTTATAAACATAGCCCATATGTATAGGCAACGCGCTGGGTACCCCGGTAC
CCGGGTGCCACTGGGCGAATGTAATTTTTATCGGCCCATAATTACCTTTAAATA
TTAGTTATCAAAACAATTCCGATGCTATTTCGGCGTGAAATGAGTTATACTCTG
GGGATCCGGAGTTTCGTGAAAAAATATTAACGGGAAGTCATTCATTTTTTCTT
TTAAACTTGAAATGGATACCCGGGTACCCGGGTGCCCAACCGAGGG

32 Kolobok1-Andl

Degenerated element

Short description

A total of 47 copies of this element were found in *An. darling* genome, the larger showing 751 bp. TIR are 14pb long, and TSD is constituted by TTAA sequence. The ORF finder found 4 short ORFs, indicating this element is not able to produce active enzymes.

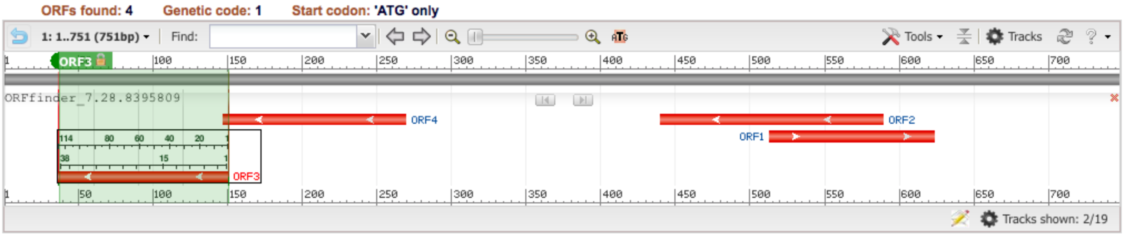

Figure 32 - Orf Finder scheme of ORFs detected in the Kolobok1-Andl element

>Kolobok1-Andl (sequence)

GGGGGGGCTTCGGTATTTTATTTTAATTCTTCGCATTTACTTTTTACATTTTTTA
TGAGTGCTTATCCTTTCAAGAGTATTGTGTAAAATTTTGGGATCAATCGAAGC
AAAACTGACAAAGATATTGATTTTTGAAAATCCGCCTTTCATACAAGGCTCAA
AGCATCTCGCAACTTTGAATCGCGATTCCCAAAACCTACGTTTTCAAAGTCG
GTGCCCATCGTAGCATAAAAACTACTGGACCGATCGATTTGAAATTTTGAACA
CATAATCTGTACACTATTTGCCAGGTAGCCCCGTCGAGTTTTTATAAAAATTGT
TGATACTTTTTTTTAAATAATTTTTTTAATTATGTAAAGTGTCGTTTTTTGAGGC
AATATCGAAAAAAGCATCACTTTTTCAACTTCAAAAATCTGCCAAAAATCGA
AAAATGGGAAATTTAACAAAAACTCGACGGGGCTACCTGGATAAACTTATAA
TCTAACAAAAGAATATTCATTTGAATATTTCTGATGACCCAGCACGTGGCTAC
GATGGGCACCGCAAAAAGTACATTTTTGCAAACTTGCCTTTCTAGATTGGATG
CCATGAACGTAGTTTTGATCAAATCTTCCCCAAAATAGAACCAAATATTCTTG
AAAAGTTGTAGTTTAATATGACATTATTTTGAAAAAATAAAGTTGAATGGTTT
CTTCACTAAAAATCGTCAAAAATAAGCCTTTTTTTGACCCGAAATAACCAAA
GCCCCCCC

33 Helitron1-Andl

Degenerated element

Short description

A total of 85 copies of this element were found in *An. darling* genome, the larger showing 1327 bp. TIR are not found, as expected. TSD consisting TTAA nucleotides. The ORF finder found 6 short ORFs.

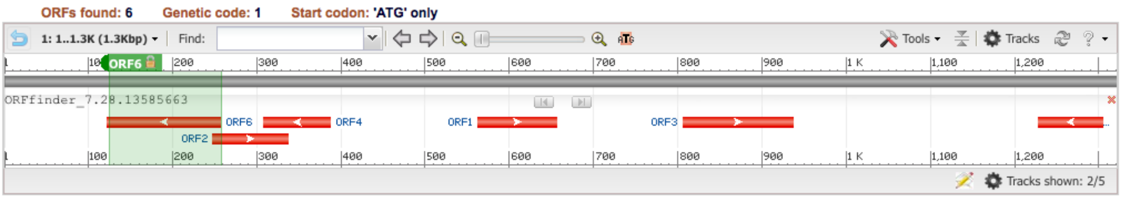

Figure 33 - Orf Finder scheme of ORFs detected in the Helitron1-Andl element

>Helitron1-Andl (sequence)

ACTAGTTGCCCCGACAGACTTCGTACTGTCTTTGATCGGTCGTGGGTTTATCG
TATACAGGAACTTTAAAATTTCACTAGATATAATTTTATCGATTCCATAATTTGG
TAGTATTCGATTTATCAAAGAACCAAATCAATAGCTAGGTCAGTTAGTGATGT
TGGAAATAGAGATACCACGTGATTGTAAATATGAACTGGAAAGTTAACATGAT
GTTTGAGTTTTAAGGCTTTGCCTTTCGGGCTAAATGACGTCATTTGGAACAAC
GAATTATATTTTCCCAAGTATTTTTAAAAAAAGTTTGACTTTAGTATTCGGCTT
GCACATAGCTCGTTAAGTTCAAATGCTGTGTATTTAACGGCATTACAATACGA
CACACTTGATCCATTTTTCCCCGGTATTCAGCTTACGATGATTTAAATAGTCAC
ATGGTAGATCATAACTAAAAGCACCGCACTTCAAATTAACTCAAACGTCGATC
GATCAGATCGAGGGTCTTTCTTTTCTCTCGGGCATTCTCGGTGATCATTCCAAT
CGCAAGTTGTTCTTGATAGATAGGATATGTTGGTATTGCTTTCTATTACTATTCG
GTTACTGCTGCTTTATATGTTTGTGCATGCAATTCCGATCGTTACCATTCATAAC
CCGATACGTTGAAAATCGTCAATGATAATAGGCACGCAAAACAATAATCAAA
AATTAATATAATTCTTGATCAATTCAAATACTTTACAACTAAATGAGATTTTATT
ATTGTTTCATGTAAATTGACTTCTGAAACTTAAAGCAACTTCTTAATTAACTAA
TGACTATCCTATCTTCCCACCAATCCAATCCAACTATCCAATTTTCCCACCCAT
CACTCTGCTCTGCAAGATGTACGGAATGGAGGGTGGTGGTGGATAGGGG
AAAGAAGCAAGTAAAAGAGTATTAAGCGAATCCAATCAATTTAGAAATCATC
AAAACCTAGGAAACGATACCCATATTGCCCTAGGATGAATTTTAAGGATTGGG
GTTGTATGGAGGCACCCCTTCCCCCTATCCGATATGAACCAAATTTTGTTCCAT
AATTTCTCAGGTGACCATCAACAACTGTGCAAGTTTTAATGGTATTCGGTTCA
TAACTTGCGCAGCTATTAATGTTTTTAACAATCAAGTGTTAGGGAAGGGCAGG
AAGGAAGGTAGCGGGGGGTTTCTAACCAAGCCTATAGCACTCACCGGCCCCT
AAAACCCTCCAATACTAAATTTGGAGTCAATTGGCCCAGTAGTTTCGGAGTCT
GTAAGGGACATCCGGACAGACATGATTCATTTTTATATATATAGA

34 Gypsy1_Andl

Putatively active element

The sequence shows a full length (5366 bp long) with LTR having 210/208 pb, and a large CDS coding the polyprotein. However, the CDS is interrupted by assemblage problems, represented by NNNNN in the sequence.

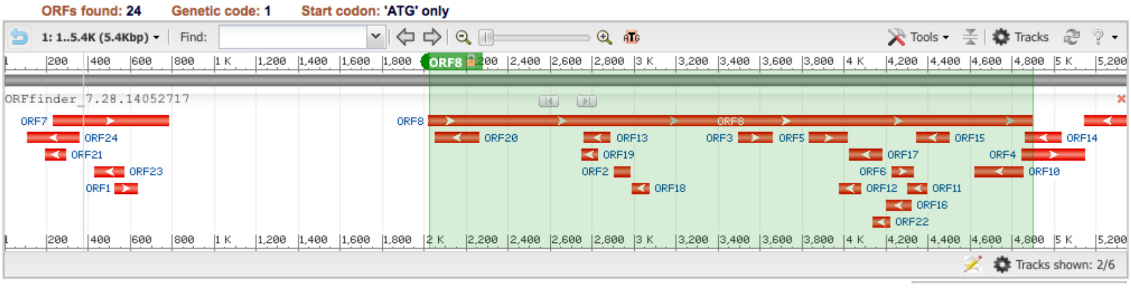

Figure 34 - Orf Finder scheme of ORFs detected in the Gypsy1_Andl element

(Blast Full=Transposon Ty3-I Gag-Pol polyprotein; AltName: Full=Gag3-Pol3; AltName: Full=Transposon Ty3-2 TYA-TYB polyprotein; Contains: RecName: Full=Capsid protein; Short=CA; AltName: Full=p24; Contains: RecName: Full=Spacer peptide p3; Contains: RecName: Full=Nucleocapsid protein p11; Short=NC; Contains: RecName: Full=Ty3 protease; Short=PR; AltName: Full=p16; Contains: RecName: Full=Spacer peptide J; Contains: RecName: Full=Reverse transcriptase/ribonuclease H; Short=RT; Short=RT-RH; AltName: Full=p55; Contains: RecName: Full=Integrase p52; Short=IN; Contains: RecName: Full=Integrase p49; Short=IN)

>lcl|ORF7
MDKKSNQPTFPEAAIASPGRTISPTGTPREQSATKNQEPQVSSSSQESAM
LQIIQMMQQQRQQQLEMFKILMKNQLETPGKTLLPIIPEQLMEMLASAMN
EFRYDAENNSTFASWYSRYEDLFHEDAERLDDAAKVRLLLRKIGQQEHER
YTSFIMPKPCRDLTFSETVATLTSLFSTKETLLK

>lcl|ORF8
MSTVGHCKRHQCRKATVSPTCASGDTLHLMGEFEAELSIEKQRRNAVIRV
SEQPQLRLLGTDLVESFELWSKPIDAFCCAISNAPSTSTTLKTVRTKFPE
IFRPELGKCTKTKVKLALKEGSTPVFCPKRPVAYAMVDAVDQELDRLEAL
GIITPIDHSEWAAPIVVVRKANGTTQICGDYSTGLNAALQPHQYPLPLPE
DIFAKLANCTVFSQIDLSDAFLQVEVDPNSRQLLSINTHCGLYQYNRLPP
GVKITPGAFQQIIDTMLANIKGVCGYMDVVVGGETESEHDKNLNAALQR
IRDYGFTIRAEKCSFGKPEIKYLGHIIDNRGLRPDPAKIEAVRELPIPSD
IAGVRSFLGAINYYGKFIENMRMLRYPLDELLKSESNFKWTNDCQKSFEK
FKEILSSNLLLTHYNPKLSIIVAADASSVGLGATISHEFPDGTRKVVQHA
SRALTKAEQNYSQPDREGLAIIFAVTKFHKMLFGRHFKLQTDHAPLLRIF
GSKKGIPVYTANRLQRFALTLLLYDFEIEYIATNKFSNADILSRLISKHT

RPEEDYVIASLKFEAEVKTIVRSAAAALPLTFEDVARKTQTDSLLRRVYN
SIKNGWESYIPDRELKPFYSRRESLSTVDGCILFGERLVIPAQYQSKCLN
QLHRGHPGIERMKAIASSYVYWPSIDDDIVQYVGSCTHCVAAAKSLTHAV
PVPWPKASGPWQRVHIDFAGPIDGDHYLVVVDSYSKWPEILRMRTISALA
TVSALRSVFARLGNPVILVSDNGTQFSSAEFAEFCANRGIKHLKTAPYRP
QSNGQAERFVDTFKRAMTKIREGRGIIIGEELDVFLHTYRSTPGAAVPDR
KSPAEVMFGRNIRTCNDLLRPPVRAKIPDLPEETMRSFSQGDAVHVKIYS
GNGWKWFPGVITARIGNVMYMMKTEKRSVKAHINQLFRREADGQHPSQCL
GFTTEYES

>Gypsy1_Andl (sequence) Red = LTRs
TGTTATGAAGGTTAGAAGATAGCCAAGGGTTTAGGGAATACAACCCTTGTGA
GAATACAACCCTTAGAATACATGCAAGAATACATCGTTTAGAGAAAAGGGAC
TATCTCAGTAACGATAGACGGAAAAAGACGTGTGGTTTGTAGGTGTGCGAAT
ATCAGTGAAATAAAGAAGTGTAAAACAACCGACTTTATTCACAATCATAACA
GTGGCGACGAGGATAAAGAATGGATAAAAAATCCAACCAACCAACGTTCCC
GGAGGCTGCGATCGCATCGCCTGGCCGGACCATATCCCCGACCGGTACGCCT
CGCGAACAGAGCGCAACGAAAAATCAAGAGCCACAAGTTTCGTCATCTAGT
CAAGAGAGTGCAATGCTGCAGATAATCCAAATGATGCAGCAGCAGCGACAG
CAACAGCTAGAAATGTTCAAAATACTAATGAAGAACCAACTTGAGACGCCGG
GTAAGACCCTTTTGCCGATAATTCCAGAGCAGCTGATGGAGATGCTGGCATCT
GCAATGAATGAGTTCCGCTACGATGCAGAAAATAATTCAACGTTTGCGTCATG
GTACAGCCGTTATGAAGATTTGTTCCACGAGGACGCAGAAAGGCTTGATGAT
GCTGCCAAAGTAAGGTTGCTATTACGAAAAATTGGCCAGCAAGAACACGAG
CGCTACACCTCGTTCATCATGCCTAAGCCCTGTCGAGATCTAACCTTTAGTGA
GACAGTAGCAACCTTAACAAGCTTATTCAGCACGAAAGAAACGCTGCTGAA
GTAAAAGATACAAGTGCCTACAACTAGAAAAACGTCCGGGAGAGGACTACA
CAGCTTACGAGTGCAGGGTCAATAAGGCATGTGTTGAATTTCAGATACGNNN
NNNNNNNNNNNNNNNNNNNNNNNNNNNNNNNNNNNNNNNNNNNNNNNNNN
NNNNNNNNNNNNNNNNNNNNNNNNNNNNNNNNNNNNNNNNNNNNNNNNNN
NNNNNNNNNNNNNNNNNNNNNNNNNNNNNNNNNNNNNNNNNNNNNNNNNN
NNNNNNNNNNNNNNNNNNNNNNNNNNNNNNNNNNNNNNNNNNNNNNNNNN
NNNNNNNNNNNNNNNNNNNNNNNNNNNNNNNNNNNNNNNNNNNNNNNNNN

NNNNNNNNNNNNNNNNNNNNNNNNNNNNNNNNNNNNNNNNNNNNNNNNN
NNNNNNNNNNNNNNNNNNNNNNNNNNNNNNNNNNNNNNNNNNNNNNNNN
NNNNNNNNNNNNNNNNNNNNNNNNNNNNNNNNNNNNNNNNNNNNNNNNN
NNNNNNNNNNNNNNNNNNNNNNNNNNNNNNNNNNNNNNNNNNNNNNNNN
NNNNNNNNNNNNNNNNNNNNNNNNNNNNNNNNNNNNNNNNNNNNNNNNN
NNNNNNNNNNNNNNNNNNNNNNNNNNNNNNNNNNNNNNNNNNNNNNNNN
NNNNNNNNNNNNNNNNNNNNNNNNNNNNNNNNNNNNNNNNNNNNNNNNN
NNNNNNNNNNNNNNNNNNNNNNNNNNNNNNNNNNNNNNNNNNNNNNNNN
NNNNNNNNNNNNNNNNNNNNNNNNNNNNNNNNNNNNNNNNNNNNNNNNN
NNNNNNNNNNNNNNNNNNNNNNNNNNNNNNNNNNNNNNNNNNNNNNNNN
NNNNNNNNNNNNNNNNNNNNNNNNNNNNNNNNNNNNNNNNNNNNNNNNN
NNNNNNNNNNNNNNNNNNNNNNNNNNNNNNNNNNNNNNNNNNNNNNNNN
NNNNNNNNNNNNNNNNNNNNNNNNNNNNNNNNNNNNNNNNNNNNNNNNN
NNNNNNNNNNNNNNNNNNNNNNNNNNNNNNNNNNNNNNNNNNNNNNNNN
NNNNNNNNNNNNNNNNNNNNNNNNNNNNNNNNNNNNNNNNNNNNNNNNN
NNNNNNNNNNNNNNNNNNNNNNNNNNNNNNNNNNNNNNNNNNNAAAATC
AAACGACAAGCATCCGCAGATAACAGAGAGCAAGGCAGTGGAGGTCAACCA
GTGCTCAGCGGAGCAACGACGAAAGTACATATTGGTCGAAATAAACACTGTT
GAATTATGTCTACAGTTGGACACTGCAAGCGACATCAGTGTCGCAAAGCAAC
CGTAAGCCCCACCTGTGCTTCGGGTGATACACTCCACCTGATGGGTGAGTTT
GAAGCTGAGTTATCAATCGAAAAACAACGACGAAATGCAGTCATCCGTGTTT
CTGAACAACCGCAACTGCGTCTGCTCGGAACGGATCTGGTAGAAAGCTTTGA
GCTCTGGTCGAAGCCAATCGATGCTTTCTGCTGCGCCATTTCCAATGCGCCCT
CTACCTCCACGACGCTTAAAACTGTAAGAACCAAGTTCCCAGAAATATTCAG
ACCGGAATTGGGAAAGTGCACAAAAACAAAGGTTAAATTGGCACTTAAGGA
AGGTTCTACCCCAGTGTTTTGCCCGAAACGTCCAGTGGCATACGCTATGGTTG
ATGCCGTGGACCAGGAGCTCGACAGGTTGGAGGCGCTAGGAATTATAACCCC
TATCGATCATTCTGAATGGGCAGCTCCAATCGTCGTTGTACGTAAAGCAAACG
GCACTACCCAGATTTGCGGGGACTACTCCACAGGGCTAAATGCCGCACTCCA
GCCACATCAATATCCACTGCCGCTACCAGAAGATATATTTGCCAAGTTAGCAA
ACTGTACGGTCTTCAGTCAAATTGACTTGTCGGATGCGTTCTTACAGGTGGA
AGTCGATCCAAACTCCCGCCAGCTATTAAGCATCAACACGCATTGCGGATTAT
ATCAGTACAATCGTCTACCACCTGGTGTAAAGATCACCCCTGGTGCTTTCCAA
CAAATCATAGACACAATGCTGGCGAACATCAAAGGTGTATGCGGTTATATGTA

CGACGTGGTAGTGGGAGGAGAAACAGAGAGCGAACATGACAAAAATCTAAA
TGCGGCTCTACAGCGAATTCGTGACTATGGATTTACAATCCGAGCTGAAAAAT
GTTCCTTCGGTAAACCCGAAATAAAATATTTAGGTCACATCATCGATAACCGT
GGATTACGACCAGATCCAGCGAAAATTGAAGCCGTCCGAGAGTTGCCAATTC
CGTCAGACATTGCGGGTGTACGCTCGTTTCTGGGAGCGATCAATTACTATGGC
AAATTTATAGAGAATATGAGAATGCTACGATACCCACTGGATGAGTTATTAAA
AAGTGAGTCCAACTTCAAATGGACTAACGACTGCCAGAAATCCTTCGAGAA
GTTTAAGGAGATTCTATCCTCCAATCTGCTGCTAACTCATTATAACCCAAAACT
GTCCATCATAGTGGCAGCAGATGCTTCATCTGTGGGATTAGGAGCGACAATCA
GCCACGAGTTCCCGGACGGAACGAGGAAAGTAGTACAGCATGCGTCGAGGG
CTCTCACGAAAGCCGAGCAGAACTATAGCCAACCGGATCGTGAAGGCTTAGC
TATTATCTTTGCAGTAACAAAGTTCCACAAAATGCTATTTGGCAGACATTTCA
AACTTCAAACAGACCATGCTCCGCTACTACGTATATTTGGTTCGAAAAAGGG
CATTCCAGTGTACACCGCTAACCGTCTTCAACGATTTGCGCTCACGCTACTAC
TATATGACTTCGAAATTGAGTACATCGCAACGAATAAGTTCAGCAATGCAGAT
ATCCTATCACGGCTTATAAGTAAACACACAAGGCCAGAAGAAGACTATGTAAT
AGCAAGTCTTAAATTTGAAGCAGAAGTCAAAACAATAGTGAGAAGCGCGGC
AGCCGCACTGCCACTAACATTCGAGGACGTAGCTCGAAAAACGCAGACGGA
CTCGCTTCTACGAAGAGTCTACAATTCTATCAAAAACGGATGGGAATCGTATA
TACCCGATCGCGAGCTAAAGCCTTTCTATTCTCGCAGAGAGTCATTGAGTACA
GTGGATGGATGTATTTTGTTCGGGGAACGACTGGTCATTCCAGCACAGTACC
AGTCGAAGTGTCTGAATCAGCTACATCGCGGACACCCGGGAATAGAACGTAT
GAAGGCCATAGCTAGCAGCTATGTGTATTGGCCTTCCATTGATGACGACATAG
TCCAGTATGTTGGAAGTTGCACACATTGTGTAGCTGCAGCCAAATCACTCAC
ACACGCAGTACCAGTGCCATGGCCTAAGGCGTCAGGCCCTTGGCAGCGTGTG
CATATCGATTTCGCTGGACCCATTGATGGTGATCATTACCTAGTGGTCGTGGAC
TCATACTCGAAATGGCCAGAGATACTACGAATGCGCACTATCAGCGCCCTTGC
TACTGTATCAGCACTCAGAAGTGTTTTTGCCAGACTAGGCAACCCTGTCATTT
TGGTCAGTGACAACGGTACTCAATTCAGCAGTGCCGAGTTCGCAGAATTTTG
TGCTAATCGAGGTATCAAACATCTGAAAACGGCCCCATACCGTCCACAATCCA
ATGGCCAAGCGGAGAGGTTTGTGGATACTTTCAAGCGGGCGATGACCAAAAT
TCGCGAGGGGAGAGGTATCATCATAGGAGAGGAACTGGACGTATTCCTACAC
ACCTACCGGAGCACCCCTGGCGCAGCAGTTCCAGATCGAAAGTCCCCTGCG
GAAGTGATGTTTGGTCGAAACATCCGCACCTGTAACGACTTACTGCGTCCAC

CAGTAAGGGCCAAGATTCCAGATCTTCCAGAAGAAACAATGAGATCCTTCTC
CCAAGGAGATGCCGTGCATGTGAAGATCTACTCTGGAAATGGTTGGAAGTGG
TTCCCGGGCGTTATCACCGCGCATCGGTAACGTCATGTACATGATGAAAAC
GGAAAAGCGATCTGTGAAGGCACATATTAACCAGTTGTTCCGTCGTGAAGCT
GATGGGCAACATCCTTCTCAGTGCCTGGGATTTACCACTGAATACGAGTCCTG
AACCTGCAGCGCAGTCGCAGTCTTCAGGATCAGCTGCGGTGGCCGAGACGA
CATCCTACCGGCCGCCCAGTTCACCAGCGAATCCCGAGCCAGCAGTGCCGTT
TCAGTCTGCAGAGCCGACTGCAGTGGCCGAGTCATCATGTCAGCACACTTCA
CCAAAGAGGCTCCACAGGCGGCGTAATCTGTCGGCAACGGCCGTCCAACCT
CGCCGTTCTTCAAGGCCCAAGAAAGCCCCTCAGCGGTTCCAGGCTTAAAGG
GGGGAGATGTTATGAGGGTTAGAAGATAGCCAAGGGTTTAGGGAATACAACC
CTTGTGAGAATACAACCCTTAGAATACATGCAAGAATACATCGTTTAGAGAAA
GGGACTATCTCAGTAAGCATAGACGGAAAAGACGTGTGGTTTGTAGGTGTGC
GAATATCAGTGAAATAAAGAAGTGTAAAACAACCGACTTTATTCACAATCATA
AAAGT

35 Gypsy2_Andl

Putatively active element

The sequence shows a full-length element with LTR having 167 pb, CDS from nucleotide 244 to 4111. However, the CDS are divided in three, which can be due to translational frameshifts that occurs in many retrotransposons. Also, the available sequence is not complete and the NNNs observed in the sequence could explain the first frameshift.

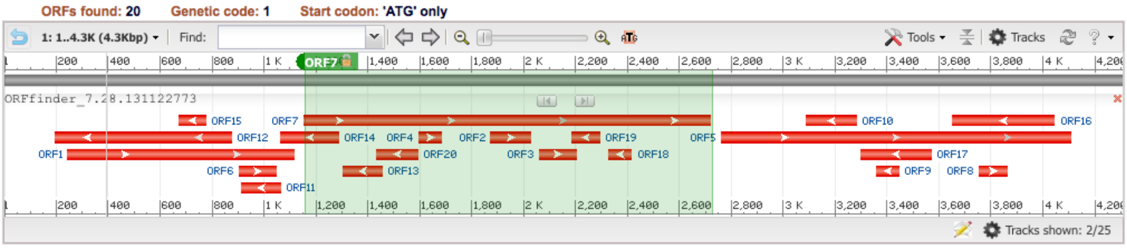

Figure 35 - Orf Finder scheme of ORFs detected in the Gypsy2_Andl element

CDS

Label	Strand	Frame	Start	Stop	Nt	aa
ORF 1	+	1	244	1119	876	291
ORF7	+	3	1152	2723	1572	523
ORF5	+	2	2762	4111	1350	449

ORF1

>lcl|ORF1 Blast (poliprotein / reverse transcriptase -Operophtera brumata)

MEPTEACGSGGRRRARGASEEVRCLDSVGKTEDKLSLDSSRVDQEAGTKM
TSIVGRPRSTTTASSVKGRRATLQLQRLEAEKAIQLEKLKLEEEFIREKF
RLMEEQLSEDDESASIENSTAQKVTSWITKSQATITSDKEGDVTREIRRM
RNELITQRPVAEEPKENAQTTSVGAEVSLWTLTKEQKAARQALPKDLPEF
NGDPADWPTFISSFNFSTKACGYQNGENMIRLQRCLKGAAKEAVRGRLLL
PESVPSVINALKRRFGRPELLLEIMIDRXXXXXXXKLVVGR

>lcl|ORF7 Blast (blastopia polyprotein - Drosophila melanogaster)
MILEEILETIGEPVVVPTEMCFLGFGGIRTKALGRITTTVCIGPEVMEMM
FYVVPRDSMGYEAILGREVLSEVEARVTQERVELVRRKVDEIEPTAETLL
CMEEEITVPTKYRKTISDLISNCVPDKTKQFESCPVELKIVPDGQLIPCR
DTPRQLAHSEEKAVDQQVEEWLQQGIVRESTSDFASKVVVVKKKDGSSRV
CVDYRKLNAGVLKDGFPVPIVEEVLNKLQKAKWFTVMDLANGFFHVPVEE
ESKKYTAFATKKGLFEFNRAPFGFCNSPAAFIRFVNYVFRDLLKKNMLDL
YMDDIVVHGETDIECLEKTKKVLETAAKAGLAVKWKKCLFLQQTITFLGH
LVENGRVSPGIEKVKAVKNFRVPKNVKGIQAFLGLTGFFRKFIKGYAEIA
RPLTDLLRKDSRFEITERELSAFNELKEQLIKEPVLRIFEQGAKTELHTD
ASKIGFGGVLMQWCDEKLHPVYFWSKKTTESESQKHSYILEAKAVFLAVK
SSEGICWVYRSSWLQIVTHSSRH

>lcl|ORF5 Blast (blastopia polyprotein - Drosophila melanogaster)
MYLQDFNFEVEHRPGTRLRHVDCLSRYPLRVMVVTSEITARIKNGQQKDE
MVKAICEILGERAYGAYHLKGGILYYAKDGQDLVVVPRGMQRQLIQEVHN
NGHFGSQKTIHALMQQYWIPQIEQKVKQTIENCVRCILYNKKLGRKEGYL
HPIGKGDKPLHTLHIDHVGPMDATSKQYRYILTMVDGFSKFVWLYPTKTT
SAEETLRKLEGWSSVFGNAERVVTDRGAAFTAHAFSEYMRINGIEHVVCT
TGVPRGNGQAERVNRTLINMLAKLSIEEPSKWFKDVPRVQRAINAHQNAT
TGKSPFELMFGVRMKNVTDNRLGEMLQQELYEEYEHDRRELREEARRAIE
EAQTGYKADFDRKRKPQVGYATGDLVAIKRTQFVAGKKLASEFLGPYEVI
KVNRNGRYKVKRVAEGEGPLITSTSEDNMKLWRYVETHAEGLSDEEEEV

>Gypsy2_AndI (sequence) Red = LTRs

ATGTAAGAATGTAAGTGTGGGCGTGAGCAAGGCAGAATGTAAGAGTGTATCG
GTGAGAGCGAATCAAGGACAAAAAGGGAAATAAAAGGAGAAATGGCGGGA
GTGCGAGGTCACTCGTGAGTTGGAGTCCCTGAGATAAAGGTGTGCAAATTAT
TTCAGTGTAACATTTGGTGTCAGAAGTGGGATTAGGATCATTATCGTGACAAG
GTGTGGTGAAAAGAACACCCTGAATAGTGATCTGTAATGGAGCCAACCGAA
GCGTGTGGTAGCGGCGGGCGGCGGCGAGCGAGAGGCGCTTCAGAGGAAGT
GCGGTGCTTGGACAGTGTCGGCAAAACGGAAGACAAACTCAGCTTGGACTC
CAGCAGAGTCGACCAAGAGGCAGGAACCAAGATGACATCGATAGTTGGGAG
GCCGAGGTCTACAACTACGGCATCGTCCGTTAAAGGAAGGCGGGCAACATTA
CAATTGCAAAGGCTCGAAGCTGAAAAAGCGATCCAGCTAGAAAAGCTGAAA
CTCGAGGAAGAGTTTATACGCGAAAAGTTTCGGCTTATGGAAGAACAGTTGT
CGGAAGATGATGAAAGTGCGTCCATCGAAAATAGTACAGCACAAAAGGTGA
CCAGCTGGATCACCAAGAGCCAGGCGACGATCACTTCGGACAAAGAAGGTG
ACGTCACGCGAGAGATTCGGCGCATGCGAAACGAGCTAATAACACAAAGGC
CGGTAGCCGAAGAACCAAAAGAAAATGCGCAAACAACAAGCGTAGGTGCA
GAAGTATCATTGTGGACGCTCACAAAAGAACAAAAGGCGGCTCGACAAGCG
CTGCCAAAAGATTTGCCGGAGTTCAACGGTGATCCCGCGGACTGGCCTACCT
TCATCAGCAGCTTCAATTTCTCCACAAAAGCATGCGGATATCAAAATGGGGA
AAACATGATCAGGCTACAACGATGCCTGAAGGGTGCTGCGAAGGAAGCAGT

GAGAGGCCGACTTCTTCTTCCAGAATCAGTTCCGAGCGTCATCAATGCGTTA
AAAAGACGTTTTGGACGGCCTGAATTGTTGCTTGAGATCATGATTGATCGNN
NNNNNNNNNNNNNNNNNNGAAAATTGGTGGTCGGCCGGTGAACGCGCTCT
TCGACACGGGTAGCCAATACAACATGATTTTGGAAGAAATATTGGAAACAAT
AGGGGAGCCGGTGGTGGTTCCAACCGAAATGTGCTTTCTTGGGTTCGGAGG
AATACGAACAAAAGCACTGGGAAGAATAACAACCACAGTGTGTATTGGGCCT
GAGGTCATGGAGATGATGTTCTATGTGGTGCCACGCGACAGTATGGGGTATGA
GGCCATACTGGGAAGGGAGGTGTTGAGCGAAGTGGAAGCAAGAGTGACAC
AGGAAAGAGTGGAGTTGGTACGGCGAAAAGTGGACGAAATCGAACCTACAG
CGGAGACATTGTTGTGCATGGAGGAAGAAATAACAGTGCCAACGAAATATCG
GAAAACAATAAGTGATCTGATTTCAAACTGCGTGCCAGACAAAACAAAACA
GTTCGAGTCGTGTCCCGTGGAATTGAAAATAGTGCCGGATGGACAATTAATTC
CATGTCGGGACACACCACGCCAACTCGCGCACTCGGAGGAGAAAGCGGTAG
ATCAGCAGGTGGAGGAATGGTTGCAGCAAGGAATCGTGCGTGAGTCAACCT
CGGACTTTGCAAGCAAGGTGGTGGTAGTTAAGAAAAAGGACGGATCAAGCC
GTGTGTGTGTGGACTATCGGAAACTGAACGCAGGGGTGTTAAAAGATGGTTT
TCCTGTGCCAATAGTAGAAGAAGTATTGAACAAGTTGCAAAAAGCTAAGTGG
TTTACAGTAATGGATTTGGCGAATGGGTTTTTTCACGTCCCGGTGGAGGAAG
AGTCGAAAAAGTACACGGCGTTCGCAACTAAAAAGGGATTATTCGAGTTCAA
TCGAGCTCCTTTCGGGTTTTGTAATTCGCCGGCGGCATTTATCCGGTTCGTTA
ATTACGTGTTTCGCGACTTGCTAAAGAAAAATATGTTGGATTTGTACATGGAT
GACATTGTGGTTCACGGCGAAACGGACATCGAGTGTTTGGAAAAAACAAAG
AAAGTGCTGGAAACCGCAGCCAAGGCCGGTTTGGCGGTTAAGTGGAAAAAG
TGTCTCTTTCTACAGCAAACGATCACTTTTCTGGGACACTTAGTCGAAAATGG
ACGGGTTTCACCGGGAATAGAGAAAGTGAAGGCAGTGAAAAATTTCCGAGT
CCCAAAAAACGTGAAAGGGATACAAGCATTTTTAGGCTTAACTGGATTTTTC
CGGAAGTTTATAAAAGGCTATGCGGAGATCGCCCGACCATTAACGGATTTACT
GAGGAAGGATAGTCGTTTCGAAATAACTGAACGGGAGTTATCGGCATTTAAC
GAGTTAAAAGAACAGTTAATAAAGGAACCTGTGCTGAGGATCTTTGAGCAGG
GAGCGAAGACGGAGCTTCATACGGACGCCTCAAAAATCGGATTTGGTGGAG
TGTTAATGCAGTGGTGCGACGAGAAGCTCCATCCAGTATACTTTTGGAGTAA
GAAAACTACCGAATCCGAATCACAAAAGCATAGTTACATCCTCGAAGCGAAA
GCGGTGTTCTTGGCGGTAAAAAGTTCAGAAGGTATTTGTTGGGTGTACCGTT
CAAGTTGGTTACAGATTGTAACGCATTCAAGCAGACACTAAGAAAAGCAGAC

GTGCCTAATGAAGTGTTACCATGGGTGATGTATCTGCAAGATTTTAATTTTGA
GGTAGAACATCGTCCTGGAACGCGACTGCGCCACGTGGATTGTCTCAGTCGG
TATCCGTTACGAGTAATGGTGGTTACATCCGAGATTACGGCGCGTATCAAAAA
CGGTCAGCAAAAGGACGAAATGGTAAAAGCTATTTGTGAAATCTTAGGAGAA
AGAGCGTATGGGGCGTATCATTTGAAAGGAGGGATTCTTTATTATGCTAAAGA
CGGACAGGATTTAGTGGTGGTTCCACGAGGAATGCAAAGGCAGCTCATTCAG
GAAGTGCATAATAACGGACATTTCGGTAGCCAGAAAACTATCCACGCTCTGAT
GCAACAGTACTGGATTCCTCAAATCGAACAAAAAGTGAAACAAACAATCGA
AAATTGCGTGAGGTGTATACTGTACAACAAGAAGTTAGGTCGTAAGGAAGGG
TATCTTCACCCCATAGGGAAGGGTGATAAGCCGCTTCACACATTGCACATCGA
TCACGTTGGACCAATGGATGCGACATCAAAGCAATACCGCTACATCCTAACA
ATGGTGGACGGATTCAGCAAGTTCGTGTGGCTGTATCCTACAAAAACTACCA
GCGCAGAGGAGACATTGCGGAAACTCGAAGGATGGTCGTCAGTGTTTGGTA
ACGCAGAACGAGTAGTGACTGATCGAGGGGCAGCATTTACAGCGCACGCGT
TTTCGGAGTATATGCGGATCAACGGGATCGAACACGTCGTCTGTACAACAGG
TGTACCGAGGGGCAATGGGCAGGCGGAGAGAGTAAACCGCACGTTGATCAA
CATGCTAGCTAAGCTCTCAATCGAAGAACCCAGCAAATGGTTTAAAGATGTT
CCCAGGGTTCAACGAGCCATTAACGCTCATCAGAATGCGACAACGGGAAAAT
CGCCATTCGAGCTAATGTTTGGGGTGCGTATGAAAAATGTAACGGACAATAG
ATTAGGAGAAATGCTACAACAGGAGTTGTATGAAGAGTATGAGCACGATAGA
CGGGAATTGCGAGAGGAAGCTAGACGAGCGATCGAAGAAGCACAGACAGG
GTACAAAGCGGATTTTGATAGGAAAAGGAAGCCGCAAGTAGGATATGCTACA
GGAGACTTAGTAGCCATTAAAAGGACACAATTTGTGGCGGGAAAAAAGTTA
GCCAGCGAATTCCTAGGGCCATACGAGGTGATTAAGGTGAACCGCAACGGGC
GGTACAAGGTCAAGAGGGTTGCGGAGGGTGAAGGACCTTTGATCACGTCAA
CAAGTGAGGACAACATGAAGTTATGGCGATATGTAGAAACCCATGCCGAAGG
GTTGTCAGATGAGGAGGAAGAGGTTTGACTAAGTGAAACGAAACATCGAGG
ATCGATGTTAGTCAGGAAAGGCCGAATGTAAGAATGTAAGTGTGGGCGTGAG
CAAGGCAGAATGTAAGAGTGTATCGGTGAGAGCGAATCAAGGCAAAAAGGG
AAATAAAAAGGAGAAATGGCGGGAGTGCGAGAACACTCGTGAGTTGGAGTC
CCTGAGATAAAGGTGTGCAAATTATTCAGTGTAACAT

36 - Copia1_AndI

Putatively active element

The element has 4291 bp and LTRs with 184 bp. The ORF expand from nt 213..3941, coding a polyprotein with 1243 aa.

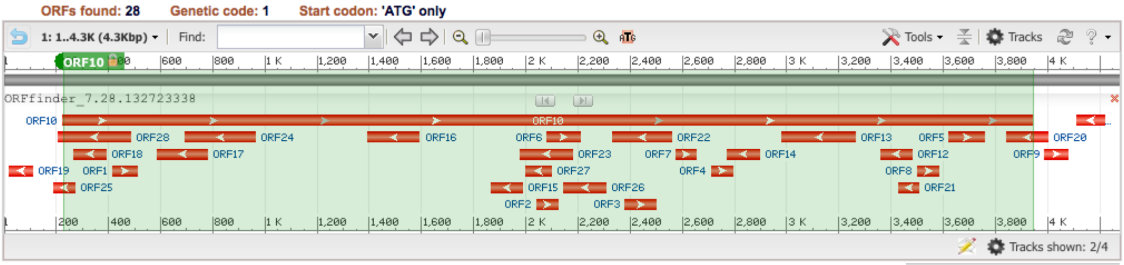

Figure 36 - Orf Finder scheme of ORFs detected in the Copia1_Andl element

CDS 213..3941

/product="Adg_Copia-70_AA-I_1 polyprotein"

KQKMDSVRVIVPRLSNDNYASWSFKVQMLLQREDVWHAVEGGKPETVTTAWT
KANTKAKAIIGLHIEDDQITLIRDCEDAKSAWEALKKFHDNVSEVYLLKKLTSLT
LAEGQSMEQHLSTFSELIQRIGASGESIPRKWQVAMLLCSLPPSYDPLTTAIELTNI
NELTVESVKSKLLAEAEKRKERAGNIESENEKAMRSEVFRRNKKPAGVVCYNC
NKPGHLKRNCRLLRNDEAKRVENNNEKPAEECAFTISQEEATEWYVDSGASRH
MTGNKEFFNQFSATNMGTVTLANGQKTRINGTGRGTLICTDSEGKPRSILLSDV
LYVPKLTSGLISVRSLTRKGMTVLFTGEQCRIKGENKEVIATASSGSLYKLHTTEK
ALKGLFQTHHEKCIHSLHRRFGHRECGVIEKMCKESGLKVKDCRIKSKCEICLK
SKMSRKPFPKKSFSETSEILQLIHTDISGPFGCTVSGYKYYMCIIDDYSRMTFLYL
LKHKSEAEDKIREFVAFCKTQIGKTPRVIRSDNGGEYTGNQLKNFLKREGIVSQL
TAPYSPQQNGVAERKNRYIQEMVRCMLADAKMEKKFWGEAALTATYIQNRLPT
TATGMTPYERWYGQKPSYDHFRIFGCEAWVHIPKERRNKMNSKAERLRFVGYS
KEQKAYRFVNEDTSKIVISRDAVFKENFNHPSWNEDILKKNKMRNQDHDIGETD
DISDVDSCVSNESDAVSDDAFEDANGVFPSESEDLPSTTDRPTRENRGKPPAYLD
DYIVGIAKSEDIEPTSWQEAMKSSHHDEWKEAMDAEMFSHEKNSTWEVTELPP

DKKAIGCRWVFKLKKDELGNVVRFKARLVAQGFNQQYGVDYDETFAPVTRFN
TIRVFLTICGRDRLIAKHLDVATAYLHGDIKEEIYMRQPPGYSLTGQGTKVCKLK
RSIYGLKQAARCWNQKLCGVLESIGFKASVTDPCLFMRIKDNSKMYLVTYVDD
LLVACKNENEIDNVYTELKKHFEINMLGDVKNFLGVQIIKNQDGILSLSMTRHID
ELALKFGLEKAKVARTPMDSGYLKCTDRQDMFEDNNKYRSIVGSLMYIANCGR
PDIAASASILGRKLECPSKTDWVAAKRVVRYLIGTRDWCLRLGDGKARCVLEV
YTDADWAGDHATRKSTTGYVLFYGGGAVAWGSRRQNCVSLSSMEAEYVAITES
CQETLWIRRLLRDLGEEQLGATFIHEDNQGCISFAQSGKISKRSKHIETRNSLSVT

> Copia1_Andl (sequence)
TGTTAAATAACAACGTTTATTTAACTCGCGTCAGGCAGCACTGCCAAAACCG
GCAGCACTGGTCAGTACTTGTTGTGTCAAGTCTTAACAACTGTGCGGTCAGT
CTTTGCATAGTTCTAGAATAAAGACCGTGCTCTGTAGAGCCAAGTGTTTTCCG
CGTTTGATTTCGACCTAAATATTAACAGGTTATGGGCCCAGCAGTGCTAACGT
GAAAACAAAAGATGGATTCGGTACGAGTAATAGTGCCAAGGTTGTCGAACG
ACAACTACGCATCGTGGAGTTTCAAGGTACAAATGCTCTTGCAGCGTGAGGA
CGTGTGGCATGCAGTGGAAGGCGGTAAGCCAGAGACAGTTACAACAGCATG
GACAAAAGCAAACACGAAGGCGAAAGCCATAATTGGTCTGCATATTGAGGAT
GACCAAATCACGCTTATCAGAGATTGTGAAGATGCAAAAAGTGCGTGGGAA
GCCCTTAAAAAGTTTCATGATAACGTATCGGAGGTGTATTTGTTAAAGAAATT
AACATCCCTCACGCTGGCAGAAGGCCAGAGCATGGAACAGCACCTGTCCAC
TTTCAGCGAGTTGATTCAAAGAATCGGTGCCTCGGGCGAATCCATTCCCAGA
AAGTGGCAGGTGGCGATGCTTCTTTGTTCACTTCCCCCCTCGTACGACCCGCT
CACAACGGCCATCGAGCTCACGAATATAAACGAGCTAACAGTGGAGAGCGT
GAAGTCGAAGTTGCTGGCAGAAGCCGAAAAGAGAAAAGAGAGGGCGGGAA
ACATCGAGAGCGAGAACGAGAAAGCAATGAGGAGTGAAGTTTTTCGCAGAA
ATAAGAAACCGGCGGGAGTCGTTTGTTATAATTGTAACAAGCCGGGACACCT
GAAGAGAAATTGCCGTCTGCTGAGAAATGATGAAGCAAAACGAGTGGAGAA
CAACAACGAAAAGCCCGCAGAAGAGTGTGCATTTACAATAAGCCAGGAAGA
AGCTACCGAATGGTACGTAGATAGTGGGGCTTCAAGGCATATGACCGGTAAC
AAGGAATTTTTCAATCAATTCTCAGCAACAAACATGGGCACAGTGACACTCG
CGAACGGCCAAAAGACCAGAATCAATGGAACTGGTAGAGGCACCCTAATATG

CACCGACAGCGAAGGAAAACCAAGATCGATCCTTCTCTCTGATGTACTTTAT
GTACCGAAACTAACAAGTGGACTAATATCCGTAAGATCTCTCACGCGAAAAG
GAATGACCGTGCTATTTACAGGTGAACAGTGCAGGATAAAAGGAGAAAATAA
AGAAGTTATAGCCACAGCTAGTAGTGGAAGTTTATATAAACTTCATACAACAG
AAAAGGCACTCAAGGGTTTATTTCAAACACACCACGAAAAATGTATTCACAG
CCTGCACAGGAGATTCGGCCACCGAGAATGTGGTGTAATAGAAAAGATGTGT
AAAGAAAGTGGATTAAAGGTAAAGGACTGCAGAATAAAATCAAAATGCGAG
ATTTGTCTAAAAAGTAAAATGTCAAGAAAACCCTTTCCGAAAAAGTCCTTTT
CAGAGACCAGCGAGATACTGCAGTTGATCCACACCGACATCAGTGGACCTTT
TGGCTGTACAGTGAGTGGATACAAGTACTATATGTGCATAATAGACGATTATAG
CAGAATGACGTTCCTATACTTGTTAAAACACAAATCGGAAGCAGAGGACAAG
ATTCGTGAGTTCGTAGCCTTCTGCAAAACGCAAATTGGGAAAACACCACGAG
TGATACGTTCCGACAATGGAGGAGAGTATACAGGAAATCAGTTGAAAAATTT
CCTGAAACGCGAAGGTATTGTCAGCCAACTAACAGCACCATATTCACCTCAA
CAAAACGGAGTCGCTGAAAGGAAAAATAGATACATACAAGAAATGGTGCGG
TGTATGCTAGCAGATGCAAAAATGGAAAAGAAATTTTGGGGCGAAGCGGCTT
TAACAGCTACGTACATACAAAACCGCTTACCCACCACAGCGACGGGAATGAC
GCCTTACGAAAGATGGTATGGACAAAAACCATCTTATGATCATTTTCGCATATT
TGGATGCGAGGCGTGGGTGCATATTCCCAAAGAGAGGCGGAACAAAATGAA
TTCCAAAGCAGAACGCCTAAGATTTGTTGGATACTCCAAAGAACAAAAAGC
ATATCGATTCGTCAACGAAGATACAAGTAAAATTGTGATCAGCCGCGATGCAG
TTTTCAAAGAAAACTTTAATCACCCTTCGTGGAATGAAGACATCTTGAAGAA
GAACAAAATGCGAAATCAAGATCATGATATCGGAGAAACCGACGACATCAGT
GACGTTGATTCGTGTGTTTCAAACGAATCAGATGCTGTGAGTGATGATGCTTT
CGAAGACGCAAATGGTGTTTTTCCTTCGGAATCTGAGGATCTACCATCAACC
ACAGATCGACCAACACGAGAAAACAGAGGGAAGCCACCAGCGTACTTGGAC
GACTATATAGTCGGGATTGCCAAGAGCGAAGATATAGAACCGACATCCTGGC
AAGAAGCGATGAAGTCATCACATCATGACGAATGGAAAGAGGCTATGGACGC
AGAAATGTTTTCACACGAGAAGAACAGCACATGGGAGGTAACCGAGCTACC
TCCTGACAAGAAAGCGATCGGATGCAGATGGGTCTTCAAATTAAAGAAAGAT
GAACTAGGAAATGTGGTACGTTTTAAGGCCAGATTGGTGGCCCAAGGTTTCA
ATCAACAATACGGTGTGGACTATGATGAAACTTTCGCACCAGTGACGCGTTT
CAACACCATTCGTGTATTCCTGACGATATGTGGAAGAGACCGTTTGATCGCCA
AACACCTTGATGTGGCTACAGCATATCTGCACGGGGACATCAAAGAAGAAAT

CTACATGCGTCAACCTCCTGGATATTCACTGACCGGACAAGGAACTAAAGTAT
GTAAGTTGAAAAGAAGTATCTACGGATTGAAACAGGCAGCGAGGTGCTGGA
ACCAGAAGTTATGTGGTGTTCTGGAAAGTATTGGATTCAAGGCTAGTGTAACT
GATCCATGTTTGTTCATGCGTATTAAAGATAATTCAAAAATGTATCTGGTCACA
TATGTTGACGACTTACTAGTAGCATGTAAGAATGAAAATGAAATCGATAACGT
ATATACAGAATTGAAGAAACACTTTGAGATAAATATGTTAGGTGATGTTAAGA
ATTTTTTAGGAGTACAAATCATCAAAAACCAGGACGGCATTCTTAGTTTGAGT
ATGACACGTCATATAGATGAACTTGCCTTAAAGTTCGGTTTAGAGAAGGCAA
AGGTGGCGCGTACTCCTATGGATTCAGGTTACCTCAAATGCACGGATAGGCA
AGATATGTTCGAAGACAATAACAAATACCGTAGCATAGTAGGTTCACTTATGT
ACATAGCAAATTGTGGAAGACCGGATATAGCAGCAAGCGCATCCATACTAGG
TAGAAAACTTGAATGTCCATCGAAAACGGATTGGGTGGCAGCGAAAAGAGT
AGTGCGTTACTTAATAGGGACACGCGACTGGTGTTTAAGATTAGGTGACGGG
AAGGCTCGATGCGTTTTAGAAGTATATACGGATGCTGATTGGGCAGGAGATCA
TGCCACGAGAAAATCTACAACAGGCTACGTCCTTTTTTATGGAGGAGGAGCT
GTTGCGTGGGGTAGTCGTCGACAAAATTGCGTTAGTCTTTCGTCGATGGAGG
CTGAATATGTAGCTATAACCGAATCATGCCAGGAAACTTTATGGATACGTAGAT
TACTGAGGGACTTAGGTGAAGAACAGTTAGGCGCCACTTTTATACACGAAGA
TAATCAAGGCTGTATAAGTTTCGCGCAATCTGGAAAAATAAGCAAGCGGTCC
AAGCACATAGAGACAAGGAATTCTTTGTCCGTGACTTAATCGACAGGAAGGA
AATTGAACTAAGGTACTGTCCTACGGAGGACATGATAGCAGACATATTAACTA
AAGCCTTAGGGAATATAAAACACAAGAAGTTCTCATTATCATTAGGACTAACG
GATCCAGTTAATTAAACGTTGAGGAGGAGTGTTAAATAACAACGTTTATTTAA
CTCGCGTCAGGCAGCACTGCCAAAACCGGCAGCACTGCCAAAACCGGCAGC
ACTGGTCAGTACTTGTTGTGTCAAGTCTTAACAACTGCGCGGTCTGTCTTTGC
ATAGTTCTAGAATAAAGACCGTGCTCTGTAGAGCCAAGTGTTTTCCGCGTTTG
ATTTCGACCTAAATATTAACA
