# Supplementary material for: A re-annotation of the Anopheles darlingi mobilome
Source: Genet Mol Biol. 2019 Jan 21;42(1):125–31. doi: 10.1590/1678-4685-GMB-2017-0300 (PMC6428116; doi:10.1590/1678-4685-GMB-2017-0300)
Supplement: Supplementary file 2 [file 1415-4757-GMB-1678-4685-GMB-2017-0300-s001.pdf]

# Supplementary Material "A re-annotation of the Anopheles darlingi mobilome"

**Table S1 -** Comparison of genome size and TEs contents in *Anopheles* genus

| Species | genome size | % of TEs | source |
|---|---|---|---|
| *A. albimanus* | 170.5 | 1.98 | 1 |
| *A. arabiensis* | 246.6 | 9.38 | 1 |
| *A. christyi* | 172.7 | 2.81 | 1 |
| *A. darlingi* | 173.9 | 2.29 / 5.61* | 2/ *3 |
| *A. dirus* | 216.3 | 5.09 | 1 |
| *A. epiroticus* | 223.5 | 6.27 | 1 |
| *A. funestus* | 225.2 | 4.03 | 1 |
| *A. gambiae* | 273.1 / 236.4 | 17.78 | 1 |
| *A. quadriannulatos* | 283.8 | 7.69 | 1 |
| *A. melas* | 227.4 / 224.2 | 7.29 | 1 |
| *A. merus* | 251.8 / 288.0 | 11.43 | 1 |
| *A. stephensis* | 221.3 / 225.4 | 5.04 | 1 |

Sources:
[1]Neafsey DE, Waterhouse RM, Abai MR, Aganezov SS, Alekseyev MA, Allen JE, Amon J, Arcá B, Arensburger P *et al.* (2015) Highly evolvable malaria vectors: The genomes of 16 Anopheles mosquitoes. Science 347:1258522.
[2]Marinotti O, Cerqueira GC, de Almeida LG, Ferro MI, Loreto EL, Zaha A, Teixeira SM, Wespiser AR, Almeida E, Silva A, Schlindwein AD, *et al.* (2013) The Genome of *Anopheles darlingi*, the main Neotropical malaria vector. Nucleic Acids Res 41:7387-7400.
[3]This study.
