# Supplementary material for: A re-annotation of the Anopheles darlingi mobilome
Source: Genet Mol Biol. 2019 Jan 21;42(1):125–31. doi: 10.1590/1678-4685-GMB-2017-0300 (PMC6428116; doi:10.1590/1678-4685-GMB-2017-0300)
Supplement: Supplementary file 3 [file 1415-4757-GMB-1678-4685-GMB-2017-0300-s002.pdf]

Supplementary Material "A re-annotation of the Anopheles darlingi mobilome"

Table S2 - Percentage of genome occupied by main TE superfamilies in studied species of the *Anopheles* genus.

	An darlingi	*An gambiae*	*An arabiensis*	*An quadriaanulatus*	*An melas*	*An merus*	*An christyl*	*An epiroticus*	*An dirus*	*An stephensi*	*An funestus*	*An albimanus*
**LTR**	**1.40**	**3.94**	**1.29**	**0.77**	**0.66**	2.56	0.08	1.22	**0.65**	**0.21**	**0.50**	**0.15**
Pao-Bel	0.06	1.60	0.57	0.29	0.24	0.97	0.01	0.65	0.23	0.05	0.12	0.01
Copia	0.74	0.40	0.18	0.15	0.12	0.28		0.07	0.06	0.04	0.13	
Gypsy	0.75	1.41	0.54	0.31	0.30	1.26	0.01	0.39	0.32	0.11	0.14	0.11
**Non-LTR**	**1.25**	**3.48**	**1.72**	**1.35**	**1.31**	**2.16**	**0.38**	**1.34**	**1.07**	**1.52**	**0.64**	**0.25**
LINE	0.15	1.67	0.63	0.49	0.78	0.85	0.01	0.16	0.57	0.12	0.03	0.03
Jockey	0.35	0.48	0.33	0.26	0.27	0.33		0.04	0.02	0.03	0.01	0.01
R1	0.37	0.24	0.01	0.08	0.08	0.15		0.06	0.06		0.08	0.08
SINE	0.11	0.43	0.51	0.45	0.47	0.21	0.39		0.08	1.34	0.36	
**DNA-transposons**	**2.44**	**1.21**	**0.73**	**0.39**	**0.42**	**0.90**	**0.10**	**0.32**	**0.26**	**0.13**	**0.14**	**0.40**
Tc1-mariner	1.06	0.59	0.48	0.21	0.22	0.29	0.03	0.22	0.12	0.10	0.06	0.28
P		0.02	0.01		0.01	0.02		0.01				
hAT	0.41	0.02	0.02	0.01	0.01	0.02	0.05		0.01	0.02		0.01
pyggyBac		0.02	0.01	0.01	0.01	0.02		0.01	0.03	0.01		
helitron	0.11	0.05		0.01	0.01	0.03						
**Total TE**	**5.67**	**17.78**	**9.38**	**7.69**	**7.29**	**11.61**	**2.81**	**6.27**	**5.09**	**5.04**	**4.03**	**1.98**

Sources:

[1]Neafsey DE, Waterhouse RM, Abai MR, Aganezov SS, Alekseyev MA, Allen JE, Amon J, Arcá B, Arensburger P *et al*. (2015) Highly evolvable malaria vectors: The genomes of 16 Anopheles mosquitoes. Science, 347:1258522. Observation: Some families were joined into superfamilies

[2]This study.
